# Cobalt‐Based Catalyst Integration Into a Hierarchically Ordered Macro‐Meso‐microporous Carbon Cathode for High‐performance Aqueous Zn‐Sulfur Batteries

**DOI:** 10.1002/advs.202509945

**Published:** 2025-08-21

**Authors:** Liangzhen Liu, Mian Zahid Hussain, Da Lei, Olivier Henrotte, Emiliano Cortés, Aliaksandr S. Bandarenka, Roland A. Fischer

**Affiliations:** ^1^ Chair of Inorganic and Metal‐Organic Chemistry Catalysis Research Center Department of Chemistry School of Natural Sciences Technical University of Munich 85748 Garching Germany; ^2^ Nanoinstitute Munich Faculty of Physics Ludwig‐Maximilians‐Universität München (LMU) 80539 Munich Germany; ^3^ Physics of Energy Conversion and Storage Catalysis Research Center Department of Physics TUM School of Natural Sciences Technical University of Munich 85748 Garching Germany

**Keywords:** aqueous Zn‐S battery, Co_3_ZnC/Co catalyst, hierarchically ordered 3D porous architecture, multistep S conversion pathway

## Abstract

The pyrolytic synthesis of an ordered macro‐meso‐micro porous carbon cathode material (OM‐PC) with integration of a Co_3_ZnC/Co catalyst is reported. It is derived from a Co‐doped ZIF‐8 framework via a templated in situ growth within the interstitial spaces of a preformed self‐assembled polystyrene monolith, followed by the template removal. The hierarchical 3D architecture facilitates Zn^2^⁺ diffusion and enhances reaction kinetics during charge–discharge processes. The integrated Co_3_ZnC/Co catalyst significantly improves the surface affinity of the porous carbon host for polysulfide trapping and accelerates polysulfide redox conversion, leading to enhanced sulfur utilization, mitigated shuttle effects, and longer cycling stability. The fabricated aqueous Zn‐S battery with the sulfur‐loaded cathode denoted as S@Co_3_ZnC/Co/OM‐PC delivers a synergistic high discharge capacity of ≈1685 mA h g−^1^, which includes ≈115 mA h g^−1^ contributed from the I_3_^−^/I^−^ redox couple. The device shows low polarization and exhibits a minimal capacity decay of ≈0.027% per cycle over 400 cycles. It maintained a good rate performance of ≈1035 mA h g^−1^ at 3 A g^−1^, with long cycling stability. In‐depth investigation reveals a multistep intermediate polysulfides conversion pathway in the aqueous electrolyte, which effectively avoids the sluggish solid‐solid conversion.

## Graphical abstract

The ordered macro‐microporous carbon material (OM‐PC) with integration of Co_3_ZnC/Co catalyst is derived from a Co‐doped ZIF‐8 via in situ growth within the interstitial spaces of a self‐assembled polystyrene monolith. The hierarchical 3D architecture facilitates Zn^2^⁺ diffusion, and the Co_3_ZnC/Co catalyst significantly improves the affinity of the carbon host for polysulfide trapping and accelerates polysulfide redox conversion in an aqueous Zinc‐sulfur battery.

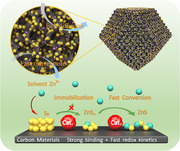

## 1 Introduction

The increasing global energy demand and alarmingly serious greenhouse gas emissions require the development of a multitude of new energy‐related technological solutions that are based on advanced materials. Here, battery technology is one highly important area. Research in sulfur (S) cathodes has been prompted, especially in nonaqueous Li‐S batteries, thanks to a high theoretical capacity (1672 mA h g^−1^), potential low manufacturing cost, and environmentally benign battery systems.^[^ ^1^ ^]^ Considering several problems of established battery systems based on non‐aqueous (organic) electrolytes, including high material cost, operational conditions, toxicity, and flammability, an alternative aqueous‐electrolyte‐based electrochemical energy storage technology appears favorable, especially for the large‐scale energy storage in stationary systems.^[^ ^2^ ^]^ Due to the natural abundance of both zinc and sulfur, aqueous Zn‐S based battery systems are being tested in large‐scale energy storage, where the Zn metal anode exhibits a high capacity of 820 mA h g^−1^ and sulfur, with an equilibrium voltage of 0.95 V, resulting in a high energy density of over 500 W h kg^−1^.^[^ ^3^ ^]^ Despite their potential, aqueous Zn‐S batteries are still in their early stages of development, facing inherent challenges.^[^ ^4^ ^]^ These include sluggish sulfur cathode kinetics, side reactions from sulfur over‐oxidation, and the parasitic hydrogen evolution reaction (HER) at the zinc anode, as well as severe zinc dendrite growth and corrosion.^[^ ^5^, ^6^ ^]^

The sulfur cathode is a vital component in metal sulfur batteries. In recent years, extensive efforts have been devoted to solving the inherent drawbacks of the sulfur cathode, such as the insulating nature of sulfur and the metal sulfides, the shuttle effect of polysulfide, and the sluggish sulfur redox kinetics.^[^ ^7^ ^]^ Strategies to resolve these challenges include encapsulating sulfur into various porous carbon materials to regulate the activities by van der Waals forces and the confined‐space effect.^[^ ^7^, ^8^ ^]^ Heteroatom‐doped carbon materials (e.g., N, S, P, B) are promising hosts for sulfur in aqueous electrolytes. Heteroatom doping modifies carbon's electronic structure and polarity, enabling moderate sulfur adsorption and enhancing conversion kinetics, while also minimizing hydrogen evolution reaction (HER) and oxygen evolution reaction (OER) activity under mild conditions.^[^ ^9^ ^]^ For instance, Li et al. reported that in sulfur and nitrogen co‐doped carbon nanofibers (denoted as S@S,N‐CNF), a composite with yolk‐shell morphology, spatially confined sulfur improves the charge transfer efficiency and lowers the activation energy, and the S and N dopants collectively catalyze the sulfur reduction reaction by accelerating the kinetics in aqueous Zn‐S batteries.^[^ ^9^ ^]^ However, the intrinsic inertness and the weak intermolecular interaction of carbon materials result in the poor long‐term cycling performance of metal‐sulfur batteries. The chemical entrapment of polysulfides (polar‐polar interaction, Lewis's acid‐based interactions, and sulfur‐chain catenation is employed to alleviate the polysulfides shuttle effects,^[^ ^10^, ^11^, ^12^, ^13^ ^]^ and highly active catalysts are introduced to immobilize polysulfides and accelerate the redox conversion reaction of the sulfur cathode.^[^ ^14^, ^15^, ^16^, ^17^, ^18^ ^]^ Recently, Zhang et al. fabricated a high‐capacity cathode by in situ interfacial polymerization of Fe^III^(CN)_6_^3−^ ‐doped polyaniline with sulfur nanoparticles. Here, the redox mediator couple Fe^II/III^(CN)_6_^4/3−^ promotes substantially faster cation (de)insertion kinetics and facilitates highly reversible conversion between S and ZnS due to the higher cathodic potential (Fe^II^(CN)_6_^4−^/ Fe^III^(CN)_6_^3−^ ≈0.8 V vs S/S^2−^), lower energy barrier and the facile Zn^2+^ intercalative transport.^[^ ^19^ ^]^ Zhao et al. designed a single‐atom cobalt catalyst, which greatly promotes the transformation of the cathode electrolyte interface (CEI) on the cathode surface and offers an accelerated transfer rate for high conversion reversibility.^[^ ^20^ ^]^

Metal‐organic frameworks (MOFs), composed of inorganic metal nodes and organic linkers, are tunable and can easily be modified; thus, MOFs and MOF derivatives are widely investigated and used in energy storage and conversion.^[^ ^8^, ^21^ ^]^ MOFs offer a highly porous framework, enabling high ionic transportation of relevant ions. However, most MOFs exhibit microporosity (the pore sizes < 2 nm), which, though they provide higher confinement ability for sulfur compared to mesoporous and macroporous carbon, restricts their applications in diffusion‐related processes.^[^ ^22^ ^]^ Recently, MOF‐based materials exhibiting hierarchical porosity, e.g., with interconnected macropores, have been developed, improving mass diffusion, electrolyte infiltration, and charge transport.^[^ ^23^, ^24^ ^]^ Cai et al. reported a 3D interconnected nitrogen‐doped hierarchical porous carbon (N‐HPC) derived from a 3D ordered macro‐microporous ZIF‐8 formulation for potassium‐ion batteries and demonstrated that the interconnected macropores of the derived carbonaceous material facilitates the diffusion of K^+^ by diminishing the diffusion distance for both ions and electrons, which improves the rate performance.^[^ ^25^ ^]^ Zhang et al. constructed a 3D‐ordered macro‐microporous carbon inlayed cobalt diselenide, which was derived from a single‐crystalline ordered macro‐microporous Co‐based MOFs (ZIF‐67). This material was used as a cathode for aluminum‐ion batteries and showed effective diffusion toward large‐sized chloroaluminate anions, an increased contact area with the electrolyte, and more exposed active sites, altogether resulting in superior reversible rate capacity and remarkable cycling performance.^[^ ^26^ ^]^ The controllable particle morphologies as well as chemical compositions and structures (topologies) of MOFs represent an attractive platform for advanced sulfur host materials. A heteroatom‐doped and hierarchically porous carbon matrix with polar/catalytic components can be designed through the judicious selection of MOF precursors and a controlled conversion process.^[^ ^27^, ^28^ ^]^

Herein, we developed a hierarchically ordered macro‐meso‐microporous carbon cathode with an integrated Co_3_ZnC/Co catalyst, labeled as Co_3_ZnC/Co/OM‐PC. The materials were derived from a hierarchically ordered macro‐mesoporous, Co‐doped ZIF‐8, labeled as OM‐ZIF‐8(Co). The material shows a good adsorption capacity for polysulfides and improves the electrical conductivity as well as hydrophilicity of the sulfur cathode, which facilitates ion transport, exposes active catalytic sites, and prevents the volume change during charging and discharging. The cathode material S@Co_3_ZnC/Co/OM‐PC exhibited high sulfur utilization, excellent rate capability (≈1651 and ≈1035 mA h g^−1^ at 0.2 A g^−1^ and 3 A g^−1^, respectively), and long‐term cycling stability with a low‐capacity decay rate (≈0.02% per cycle within 400 cycles at 3 A g^−1^). The uniformly embedded Co_3_ZnC/Co catalysts show a high catalytic conversion activity toward polysulfides (PSs), which efficiently inhibits the shuttle effect of PSs. Evident from the results, the specific benefits of the detailed architectural and chemical features of Co_3_ZnC/Co/OM‐PC for the adsorption and transformation of sulfur in aqueous Zn‐S batteries were demonstrated.

## 2 Results and Discussion

### 2.1 Materials Synthesis and Characterization

**Figure 1:**
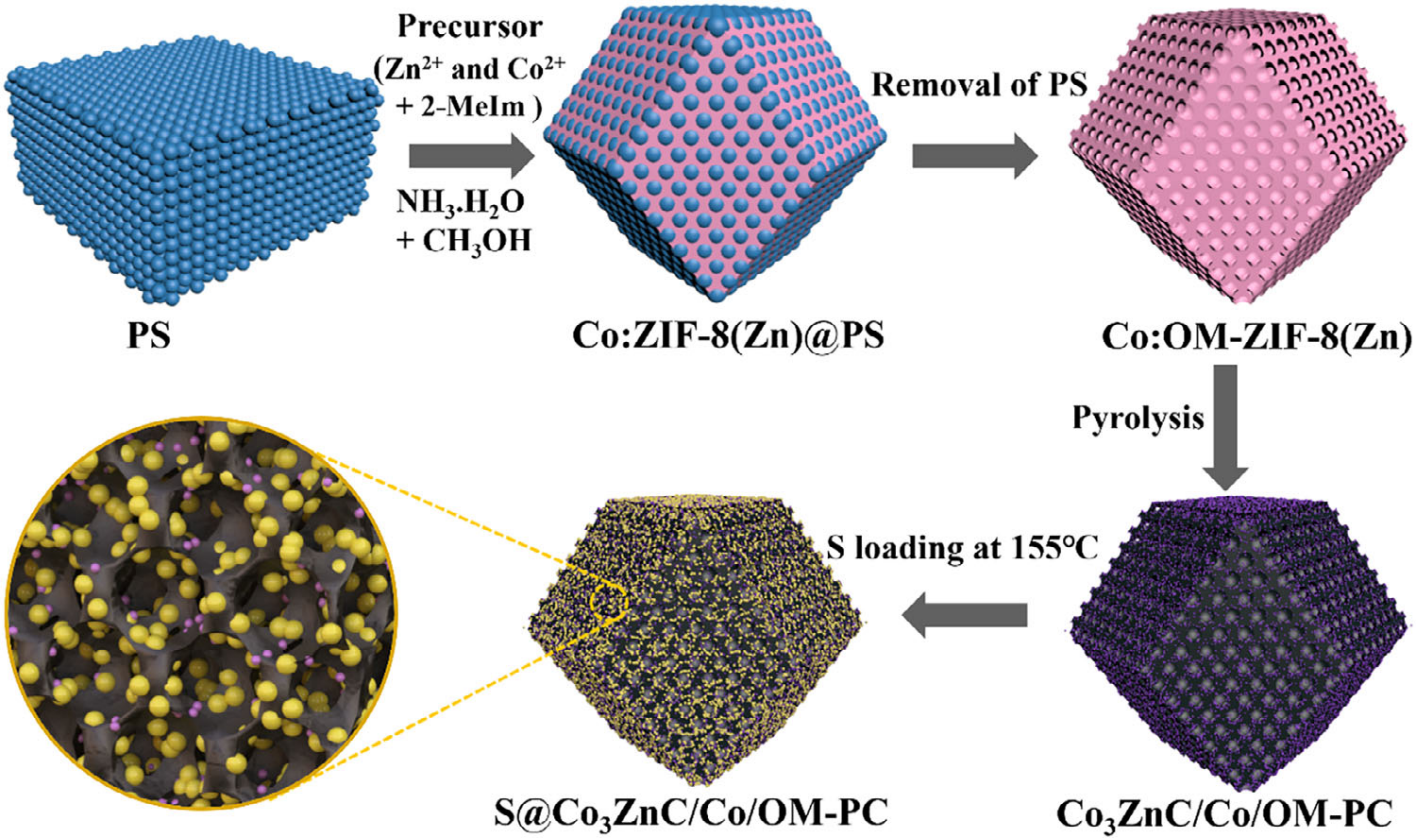
Schematic diagram of the synthetic procedure of the cathode material for the aqueous zinc‐sulfur battery, denoted as S@Co_3_ZnC/Co/OM‐PC. (Blue: PSs template; Pink: ZIFs crystal; Black: carbon framework; Purple: Co_3_ZnC nanoparticles; Yellow: S nanoparticles).

As a subclass of MOFs, zeolitic imidazolate frameworks (ZIFs) exhibit tunable porosity, high surface area and exceptional mechanical stability, resulting from the metal‐nitrogen bonds, making ZIFs promising precursor materials to thermally derive various N‐doped porous carbon‐based composites in which the inherited nitrogen from organic linker can provide catalytic sites, enhance electron transfer rate, and interact with electrolytes and reactants.^[^ ^29^, ^30^ ^]^ MOFs can be formulated into macroscopic shapes, which contain macropores via an external‐template method. This allows the fabrication of an overall hierarchically ordered macro‐meso‐microporous monolith structure based on heterogeneous nucleation approaches.^[^ ^31^ ^]^ A schematic diagram in **Figure** **1**a illustrates the fabrication procedure of the final target material Co_3_ZnC/Co/OM‐PC by a polystyrene (PS) monolith as an external template. This template features PS beads with a diameter of ca 300 nm, which self‐assembles in a fcc packing mode (Figure S4, Supporting Information). The 3D ordered macro‐meso‐microporous ZIFs (Co:OM‐ZIF‐8(Zn) and OM‐ZIF‐8(Zn)) (Figure S5a,b, Supporting Information) exhibits a polyhedral morphology with a well‐defined 3D‐ordered macroporous architecture, distinct from conventional ZIF‐8(Zn) (Figure S5c, Supporting Information). These materials were synthesized through the in situ growth of ZIFs within the interstitial voids of a highly ordered PS monolith, followed by PS removal in a CH_3_OH/NH_3_·H_2_O solution. The powder X‐ray diffraction (PXRD) analysis (Figure S6, Supporting Information) revealed that Co:OM‐ZIF‐8(Zn) and OM‐ZIF‐8(Zn) exhibit diffraction patterns identical to those of conventional ZIF‐8(Zn), confirming their high crystallinity and structural integrity.

**Figure 2:**
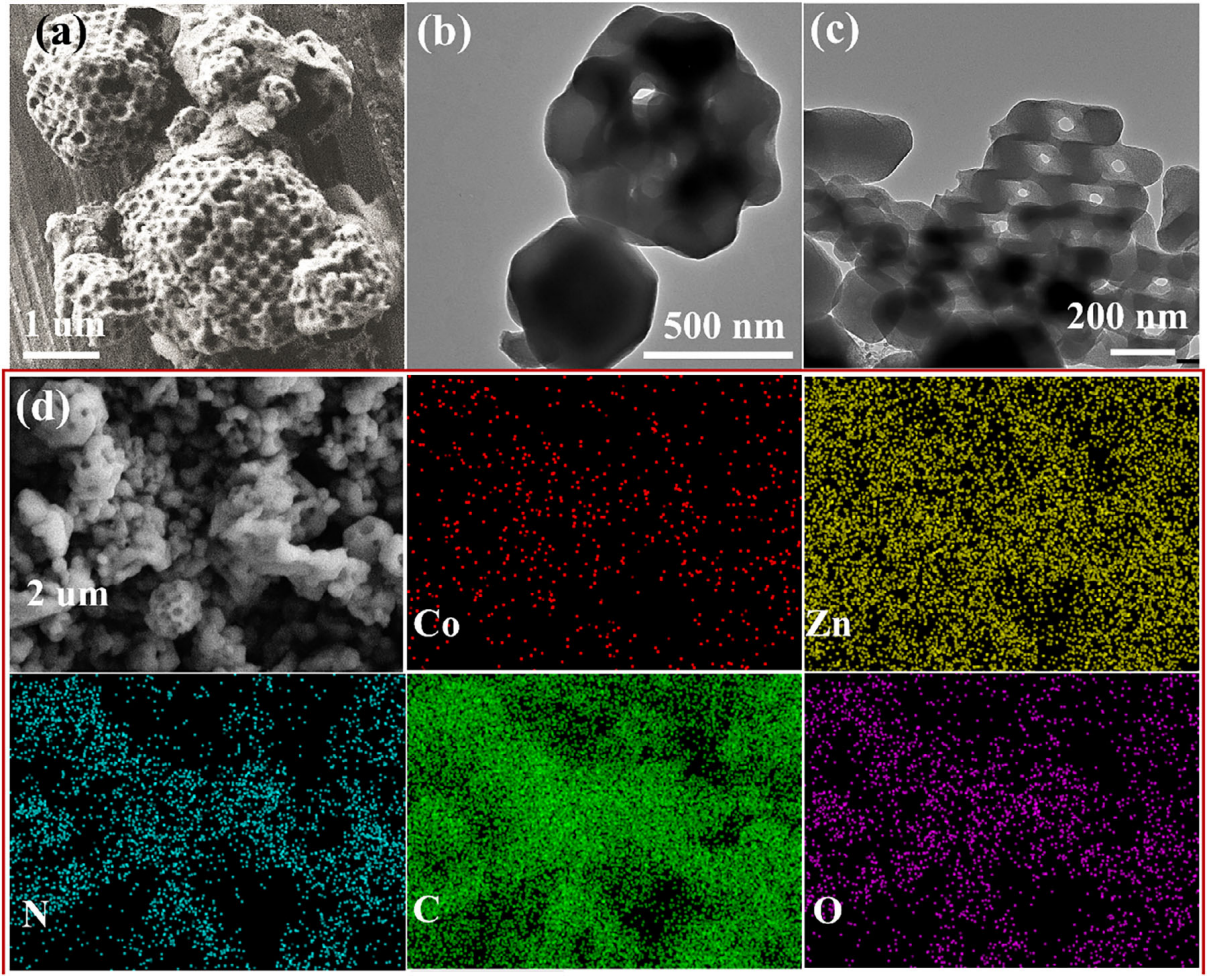
a) SEM micrograph; b,c) TEM images; and d) the SEM/EDS elements maps of as‐synthesizedCo_3_ZnC/Co/OM‐PC.

The scanning electron microscopy (SEM) images confirm that the Co_3_ZnC/Co/OM‐PC (**Figure** **2**a) and OM‐PC (Figure S7a, Supporting Information) preserve the tetrahedral morphology and highly ordered microporous structure of the original template Co:OM‐ZIF‐8(Zn) and the OM‐ZIF‐8(Zn) composite after pyrolysis. This special morphology significantly differs from conventional porous carbon (PC) materials (Figure S7b, Supporting Information). The interconnected macropores, which are formed during pyrolysis because of the initial PSs templating approach, can provide large channels to expedite diffusion of larger molecules, even macromolecules, and facilitate electrolyte infiltration.^[^ ^26^ ^]^ The transmission electron microscopy (TEM) (**Figure** 2b,c) and SEM results show the interconnected hierarchically macro‐microporous framework in Co_3_ZnC/Co/OM‐PC. The elemental mapping (EM) (**Figure** 2d) further confirms the uniform distribution of Co, Zn, C, and N elements in the Co_3_ZnC/Co/OM‐PC.

**Figure 3:**
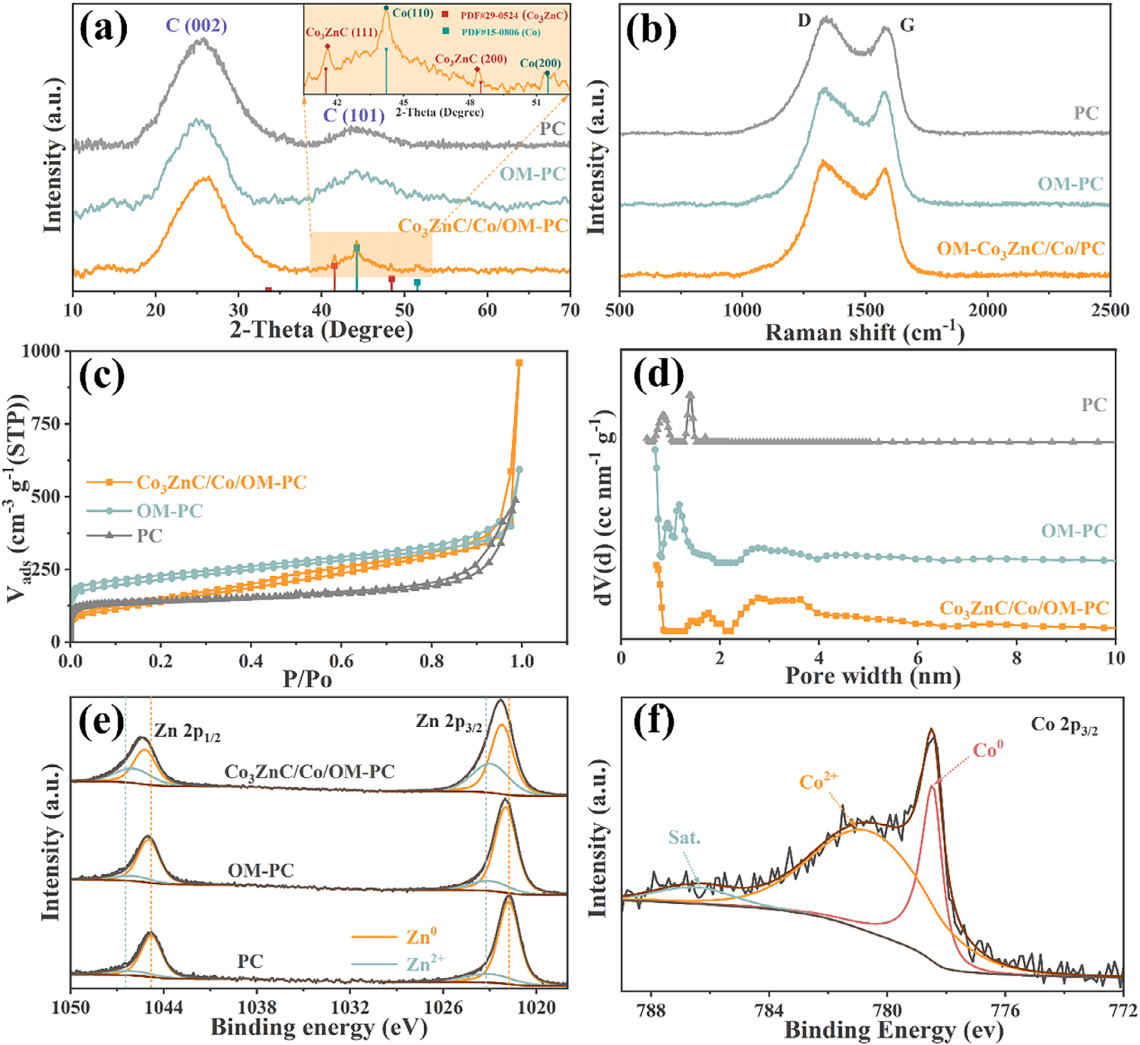
a) PXRD patterns; b) Raman spectra; c) N_2_ adsorption‐desorption isotherms; d) the pore size distributions; the high‐resolution XPS of e) Zn 2p of the three host materials and f) Co 2p of Co_3_ZnC/Co/OM‐PC.

The phase analysis of the target material was done by standard powder XRD. As shown in **Figure** **3**a, the peaks at 43.6° and 48.5° correspond to the (111) and (200) crystalline planes of Co_3_ZnC, while the peaks at 44.2° and 51.6° are attributed to the (111) plane of Co and the (200) plane of C, respectively. These observations confirm the formation of Co_3_ZnC and Co nanoparticles during pyrolysis.^[^ ^32^ ^]^ The templated MOF precursors transformed into graphitic carbon, with most zinc volatilizing at the high pyrolysis temperatures applied. Consequently, the XRD patterns exhibit peaks at ≈26.0° and 44.0°, corresponding to the (002) and (101) planes of graphitic carbon, respectively.^[^ ^33^ ^]^ The carbon component was further characterized by Raman spectroscopy (**Figure** 3b). It confirms a highly graphitized carbon support, and the typical D band position of carbonaceous (graphitic) materials is seen at ≈1330 cm^−1^, that stems from the defects in the graphitic lattice, and the G band originating from internal defect‐induced scattering at 1579 cm^−1^, respectively.^[^ ^32^ ^]^ The I_D_/I_G_ intensity ratio of Co_3_ZnC/Co/OM‐PC allows for the evaluation of structural disorder and the estimation of the average in‐plane crystallite size of the sp^2^ domain. The value is ≈1.08, which illustrates the presence of abundant defect sites in Co_3_ZnC/Co/OM‐PC.

The specific surface area (SSA) (**Figure** 3c) and pore size distribution (**Figure** 3d) were estimated by standard N_2_ sorption isotherms. The N_2_ sorption isotherms of Co_3_ZnC/Co/OM‐PC, OM‐PC, and PC show a type‐IV isotherm, confirming the presence of micropores and mesopores. The SEM micrograph (**Figure** 2a) directly confirms the presence of macropores. The BET specific surface areas were determined by standard nitrogen adsorption studies to be ≈720, ≈770, and ≈530 m^2^ g^−1^ for Co_3_ZnC/Co/OM‐PC, OM‐PC, and PC, respectively. The pore size distribution ranging from 0.5 to 5.0 nm in Co_3_ZnC/Co/OM‐PC and OM‐PC confirms the macro‐ and microporous structure of interconnected channels.

X‐ray photoelectron spectroscopy (XPS) was employed to further characterize the chemical nature of the components. A survey spectrum in (Figure S8a, Supporting Information) shows the presence of Co, Zn, N, O, and C elements in the Co_3_ZnC/Co/OM‐PC. The deconvoluted C 1s peaks (Figure S8b, Supporting Information) of PC can be assigned to C─C at 284.8 eV, C─N/C─O at 285.8 eV, C═O at 287.6 eV, and O─C═O at 289.1 eV, respectively. Compared to the PC, Co_3_ZnC/Co/OM‐PC and OM‐PC exhibited two additional peaks at 248.4 and 291.5 eV, which may arise from the C═C bond and π–π derived from the residual species of polystyrene.^[^ ^34^ ^]^ These results prove the formation C─N bonds during carbonization in all samples. Such Lewis base “catalyst” offers stronger entrapping capacity to polysulfides and a higher number of electroactive sites, which improves the oxidative conversion of S_6_^2−^ to S_8_. Regarding the N 1s spectra for Co_3_ZnC/Co/OM‐PC, it features three peaks (Figure S8c, Supporting Information), including pyridinic‐N and/or metal‐N coordination (Co─N) at 398.8 eV, graphitic‐N at 401.3 eV, and oxidized‐N species at 402.5 eV. Whereas, for OM‐PC and PC, the N 1s binding energies were centered at 398.4 eV (pyridinic‐N), at 400.2 eV (pyrrolic‐N), and oxidized‐N species at 402.5 eV, respectively.^[^ ^35^ ^]^ Pyrrolic‐N and pyridinic‐N species have higher affinities to sulfur species, while the Co─N binding offers more active sites for sulfur entrapment, i.e., p‐type doped pyridinic N contains two p‐electrons that can coordinate sulfur atoms.^[^ ^36^ ^]^ Thus, the high N content (approximately up to 23 wt.%) enhances electron transfer and at the same time serves as active sites for improving electrochemical performance. The Zn 2p spectra shown in **Figure** 3e contains the contribution of both to Zn 2p_3/2_ and Zn 2p_1/2_. For Zn^0^, the binding energies centered at ≈1021.80 and 1044.81 eV, and at 1023.29 and 1046.35 eV for Zn^2+^ in PC and OM‐PC, respectively.^[^ ^37^ ^]^ Interestingly, the Zn^0^ peak in OM‐Co_3_ZnC/Co/PC shifted positively by ≈0.4 eV compared to PC and OM‐PC, Additionally, the Zn^2+^ peak in Co_3_ZnC/Co/OM‐PC shifted negatively by ≈0.27 eV compared to PC and OM‐PC. Notably, the Zn^2+^ content in Co_3_ZnC/Co/OM‐PC (≈38.25 at. %) doubled compared to PC (≈17.89 at. %) and OM‐PC (≈16.32 at. %) to ≈38.25 at.%. This result suggests a better electron transfer capability of Zn, potentially resulting from the increased loading and uniform nano‐dispersion of Zn (i.e., Co_3_ZnC nanoparticles) within Co_3_ZnC/Co/OM‐PC.^[^ ^38^ ^]^ As shown in Figure 3f, the high‐resolution Co 2p spectrum can be deconvolved into three peaks corresponding to the Co^0^ peak at 778.4 eV, the Co^2+^ peak at 781.05 eV, and a satellite peak at 784.5 eV. The derived relative ratio of Co^2+^/Co^0^ is ≈2.4. This ratio can chemically bind intermediate polysulfides due to the Lewis acidic Co (II) character. The electron transfer between Co^0^ and Co^2+^ may also have a cooperative effect on electrocatalytic activity.^[^ ^15^, ^35^ ^]^ Thus, the Zn^2+^‐modulated bimetallic carbide electrocatalyst not only contributes to entrapping polysulfides effectively but also accelerates the catalytic conversion kinetics of polysulfide intermediates.

### 2.2 Polysulfides Trapping Mechanism

**Figure 4:**
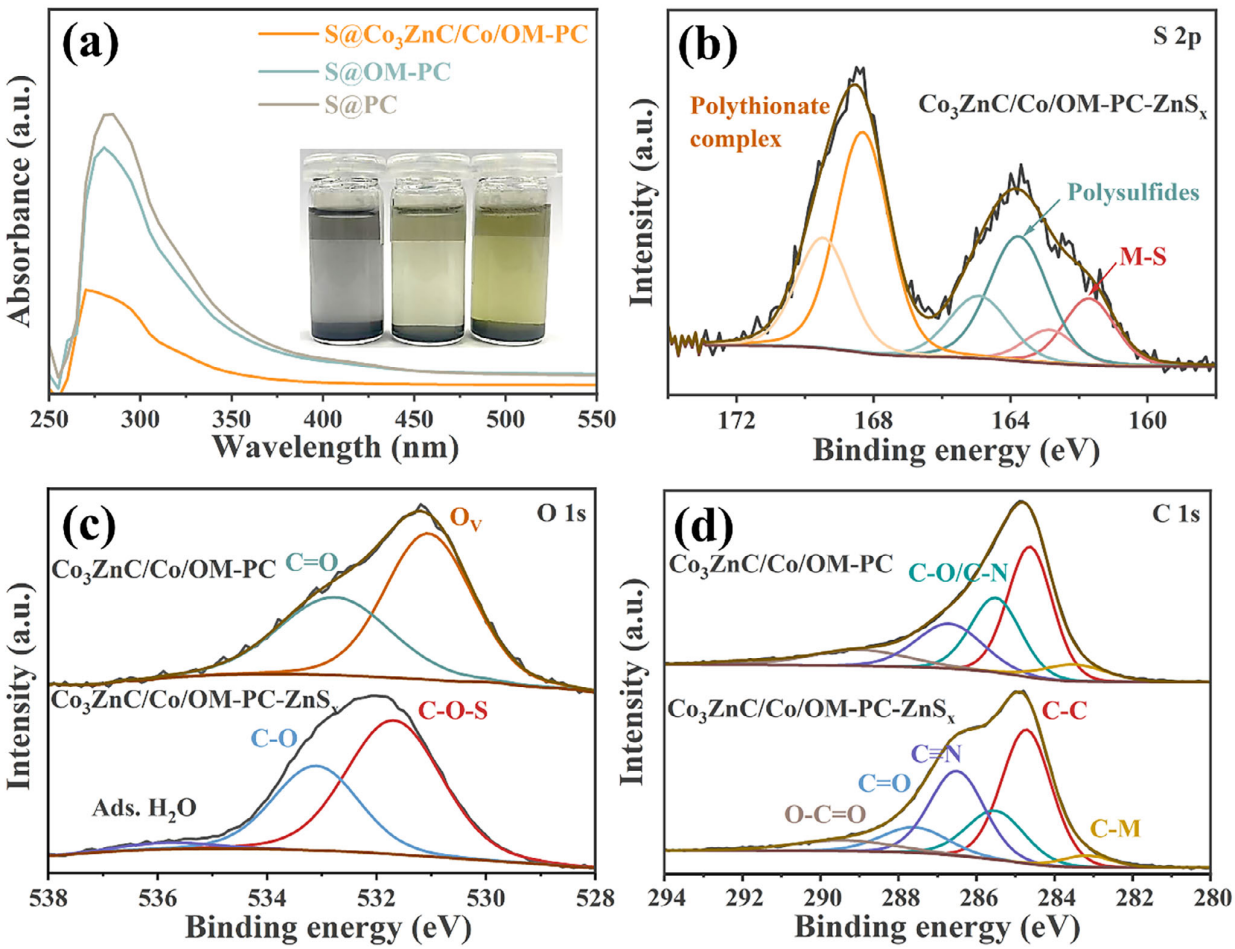
a) The UV–vis spectra of the polysulfides solution after adsorbing by Co_3_ZnC/Co/OM‐PC, OM‐PC and PC. (Insert: Optical images of polysulfides solutions after adsorbing by different host materials). The high‐resolution XPS spectra of b) S 2p, c) O 1s, and d) C 1s of Co_3_ZnC/Co/OM‐PC and Co_3_ZnC/Co/OM‐PC‐ZnS_x_.

To further corroborate the enhanced affinity of Co_3_ZnC/Co/OM‐PC toward polysulfides, adsorption experiments were performed by adding Co_3_ZnC/Co/OM‐PC, OM‐PC, and PC into polysulfides solution. As shown in **Figure** **4**a, the polysulfides solution treated with Co_3_ZnC/Co/OM‐PC became colorless after 12 h, whereas the color of the other two solutions remained yellowish. The polysulfides solution with Co_3_ZnC/Co/OM‐PC exhibited the lowest absorbance in the UV–vis absorption spectra, suggesting the relatively strongest affinity of Co_3_ZnC/Co/OM‐PC toward polysulfides. To further investigate the chemical interactions between Co_3_ZnC/Co/OM‐PC and polysulfides, XPS spectra were performed on Co_3_ZnC/Co/OM‐PC pre‐soaked in a polysulfide solution. The S 2p_3/2_ signals of Co_3_ZnC/Co‐ZnS_x_ (Figure 4b) at 161.7 and 162.9 eV can be assigned to the terminal S‐Co/Zn bonds, suggesting metal‐sulfur (M‐S) interaction.^[^ ^39^, ^40^ ^]^ The metastable Co centers may receive electron density from polysulfide anions, forming Co‐S bonds via the Lewis acid‐base interaction.^[^ ^40^ ^]^ Meanwhile, the S 2p_3/2_ peaks at 163.7 and 164.9 eV can also be assigned to polysulfides.^[^ ^40^ ^]^ Two additional peaks (different from polysulfides and M‐S) were also observed at 168.3 and 169.5 eV, indicating the formation of polythionates.^[^ ^41^, ^42^ ^]^ Accordingly, the generation of C─O─S bonds (**Figure** 4c) contributes to the adsorption of the polysulfides.^[^ ^43^ ^]^ Furthermore, the assignment of the C─M bonds at 283.3 eV in C 1s peak (**Figure** 4d) suggests that the stability of the carbide and the formation of M‐S may originate from the oxidation layer at the surface of carbides.^[^ ^44^, ^45^ ^]^ The formation of polythionate intermediates, Co─S bonds and C─O─S, may facilitate the conversion of soluble polysulfides and alleviate shuttle‐related issues.

### 2.3 Electrochemical Kinetics and Mechanism

#### 2.3.1 Diffusion Kinetics Mechanism

**Figure 5:**
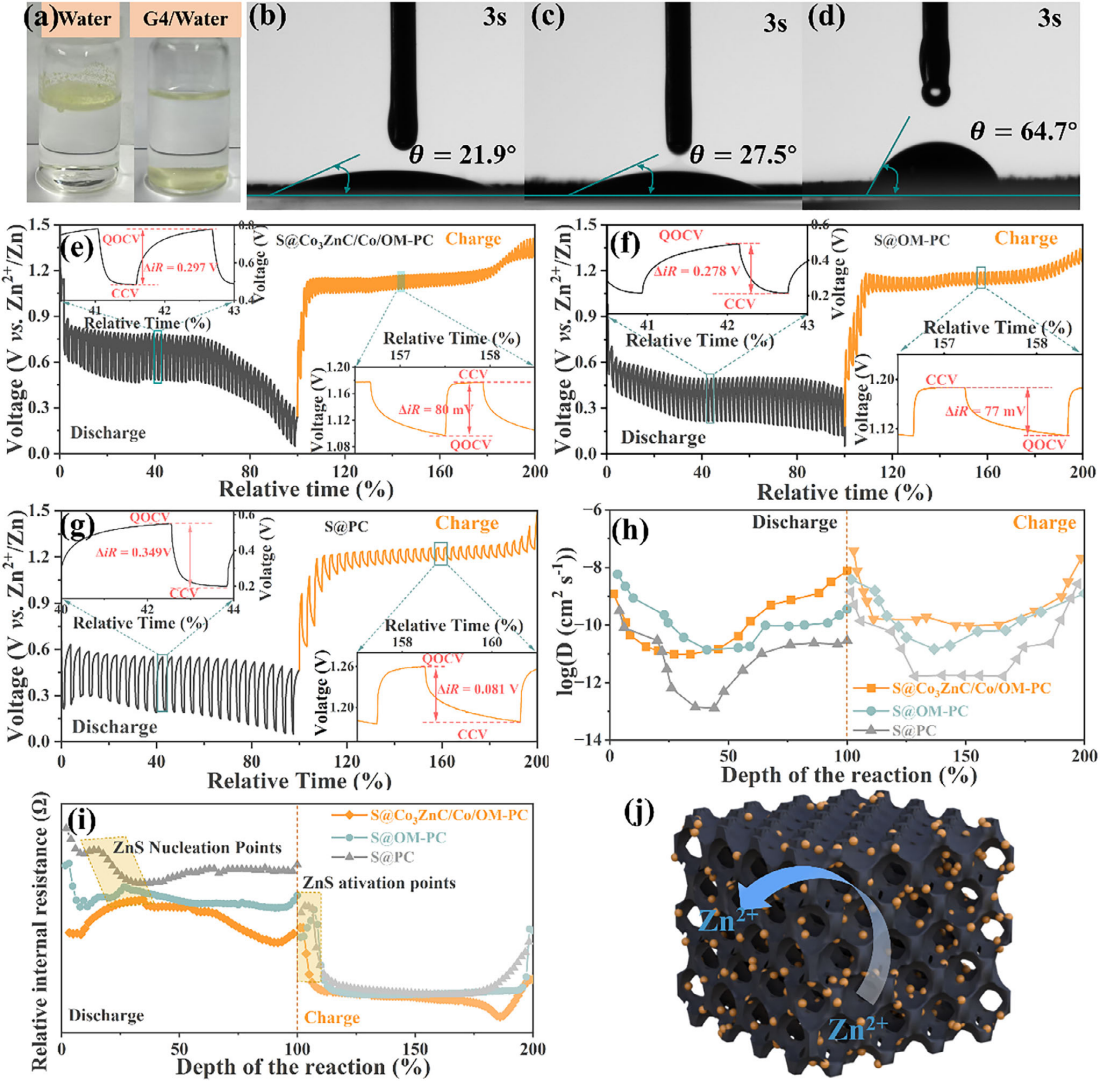
Photographs of a) sulfur powder immersed in deionized water (left) and the mixture of G4 and water (right), and the static contact angles of electrolyte on b) S@Co_3_ZnC/Co/OM‐PC electrode, c) S@OM‐PC electrode, and d) S@PC electrode, respectively. The GITT plots of aqueous Zn‐S cells for e) S@Co_3_ZnC/Co/OM‐PC electrode, f) S@OM‐PC electrode, and g) S@PC electrode. h) the diffusion coefficients of Zn^2+^ ions and i) internal resistance with respect to normalized discharge–charge time were calculated from GITT. j) The diagram of the zinc ions migrating into the 3D interconnected hierarchical porous framework.

In this study, the sulfur loading in the cathode materials was ≈50 wt.%, as confirmed by TGA (Figure S9a, Supporting Information) and XRD (Figure S9b, Supporting Information) analyses. The XRD patterns of S@Co_3_ZnC/Co/OM‐PC, S@OM‐PC, and S@PC clearly exhibit the sulfur peak. The electrochemical performance of the prepared S cathodes was evaluated in aqueous zinc cells against zinc foil as the anode. The Zinc trifluoromethanesulfonate (Zn(OTf)_2_) was used as the zinc salt, water and tetraethylene glycol dimethyl ether (G4) served as the co‐solvents, and ZnI_2_ (introducing the I_3_^−^/I^−^ redox couple) was used as the electrolyte additive, respectively. The wettability of the electrolyte is one of the important factors influencing the electrochemical performance of the S cathode. As shown in **Figure** **5**a, sulfur powder can be soaked in the mixture of G4 and water. However, in pure water, it has the opposite behavior. Thus, G4 can greatly improve the wettability of sulfur and improve sulfur dispersion in the electrolyte.^[^ ^46^ ^]^ It is concluded that the 3D ordered macroporous structure and the interconnected pores in S@Co_3_ZnC/Co/OM‐PC facilitate mass diffusion, the electrolyte infiltration into the cathode material, and the charge transport.^[^ ^47^ ^]^ In order to quantify the effect of 3D ordered macropores on electrolyte wettability, contact angles were measured for the three sulfur cathodes. As shown in **Figure** 5b–d and S@Co_3_ZnC/Co/OM‐PC and S@OM‐PC electrodes exhibited excellent wettability, with instant wetting upon electrolyte contact. In contrast, the S@PC electrode showed poor wettability, with a relatively higher contact angle of 64.7°.

Galvanostatic intermittent titration technique (GITT) measurements were conducted using current pulses of 0.1 A g−^1^ for 15 min, followed by a 0.5‐h relaxation period. From the data, one can extract information about the Zn^2^⁺ diffusion kinetics, i.e., the diffusion coefficient ($D_{Zn^{2+}}$) in the 3D interconnected macroporous structure. As shown in **Figure** 5e–g, the potential change during each relaxation period represents η_overpotential_ at the corresponding reaction stage. Compared with S@PC and S@OM‐PC electrode, S@Co_3_ZnC/Co/OM‐PC electrode exhibited lower overpotentials, meaning smaller IR drops (**Figure** 5h) during discharge and charge processes. This indicates relatively smaller internal resistance. The value of $D_{Zn^{2+}}$ was calculated according to the GITT measurements using Equation S5 (Supporting Information). The calculated result is plotted as a function of the depth of the discharge and charge. As shown in **Figure** 5i, the S@Co_3_ZnC/Co/OM‐PC and S@OM‐PC electrodes display high $D_{Zn^{2+}}$ value than S@PC electrode at all charge‐discharge process, indicating faster Zn^2^⁺ diffusion kinetics. This enhancement is attributed to their 3D hierarchically ordered macro‐meso‐microporous structures, as illustrated in **Figure** 5j. The uniform open macropores in S@Co_3_ZnC/Co/OM‐PC and S@OM‐PC enhance electrolyte penetration, shortening mass diffusion paths and facilitating Zn^2^⁺ diffusion kinetics, thereby improving the electrochemical performance of S electrode in aqueous Zn‐S batteries.^[^ ^22^, ^48^ ^]^

#### 2.3.2 Electrochemical Activity for Polysulfides Conversion Mechanisms

**Figure 6:**
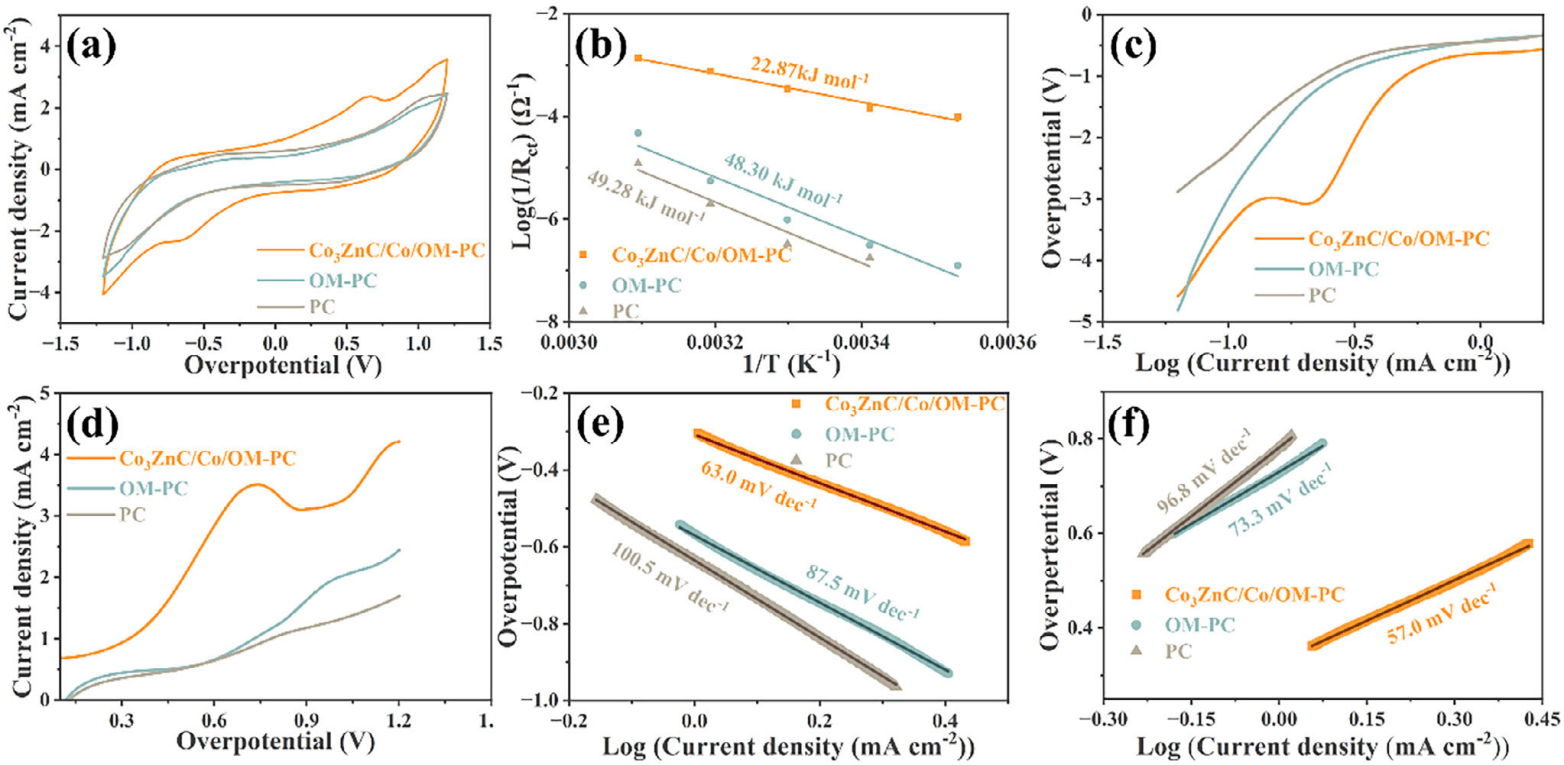
Electrochemical performance of symmetric batteries (with identical electrodes) and electrolyte with ZnS_x_. a) CV curves of symmetrical cells at a scan rate of 5 mV s^−1^; b) the comparison of activation energies of Co_3_ZnC/Co/OM‐PC, OM‐PC, and PC derived from corresponding Arrhenius curves. c,d) Linear sweep voltammetry (LSV) curves of Co_3_ZnC/Co/OM‐PC, OM‐PC. and PC of the polysulfide reduction reaction process (ZnS_x_ → ZnS_n_) and oxidation reaction process (ZnS _n_ → ZnS_x_). Tafel plots for e) polysulfide reduction reaction process and f) polysulfide oxidation reaction process.

The symmetric cells with identical electrodes and the ZnS_x_ electrolyte were assembled to investigate the conversion kinetics of polysulfides. It is apparent that Co_3_ZnC/Co/OM‐PC exhibits distinct redox peaks with the highest peak current density, lower polarization potential, and small initial overpotential compared with OM‐PC and PC, suggesting the superior catalytic activity of Co_3_ZnC/Co/OM‐PC for efficient polysulfides redox reaction kinetics (**Figure** **6**a). The enhanced kinetics of polysulfide conversion are further verified by the activation energy (E_a_) (**Figure** 6b) calculated from charge transfer resistance (R_ct_) of Nyquist plots of symmetric cells at different temperatures (Figure S10, Supporting Information),^[^ ^5^ ^]^ demonstrate the smallest E_a_ value for the redox reactions of polysulfide facilitated by Co_3_ZnC/Co/OM‐PC. Moreover, the Tafel slopes derived from linear sweep voltammetry (LSV) curves (**Figure** 6c,d) are calculated to testify the kinetics of the polysulfide oxidation process and reduction reaction process. The smallest Tafel slopes for the reduction and oxidation processes are observed for Co_3_ZnC/Co/OM‐PC (**Figure** 6e,f), validating the effectively bidirectional catalytic conversion of ZnS_x_ and ZnS_n_.

### 2.4 Aqueous Zn‐S Batteries Electrochemical Properties and Sulfur Conversion

#### 2.4.1 Electrochemical Performance Properties

**Figure 7:**
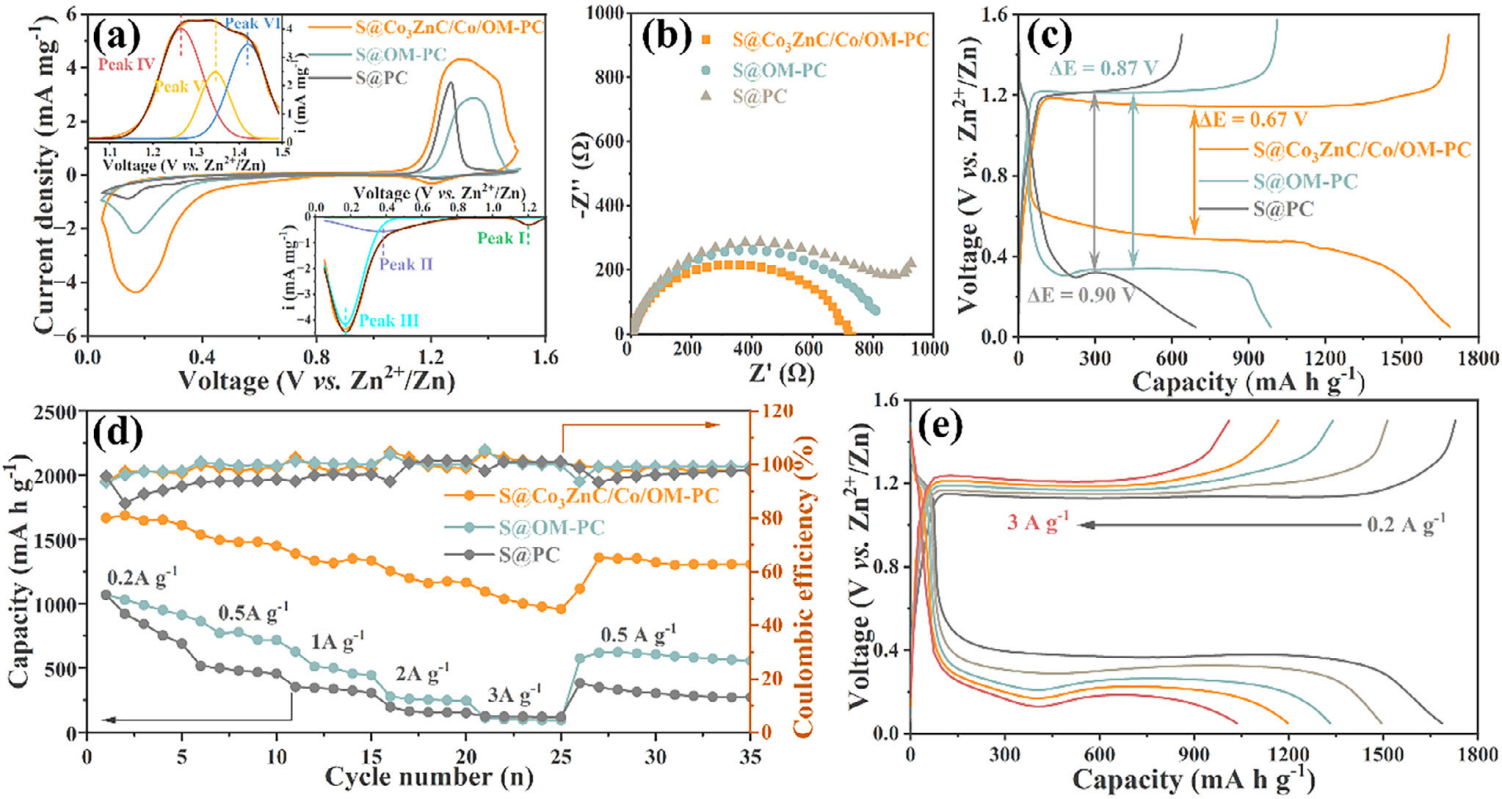
Electrochemical performance of aqueous Zn‐S batteries. a) the third cycle of CV curves of different S electrodes at 0.1 mV s^−1^;(Insert image: the deconvoluted peaks of CV curve of S@Co_3_ZnC/Co/OM‐PC) b) EIS curves of different S electrodes; c) GCD profiles of different S electrodes; d) Rate performance; e) the GCD curves of S@Co_3_ZnC/Co/OM‐PC cathode at current density from 0.2 to 3 A g^−1^.

The sulfur conversion behavior of these three electrodes was initially assessed via cyclic voltammetry (CV) over a potential range of 0.05–1.5 V (vs Zn/Zn^2^⁺) at a scan rate of 0.1 mV s−^1^. As shown in **Figures** **7**a and S11 (Supporting Information), cathodic Peak I at ≈1.20 V is attributed to the redox reaction of iodine species (I^−^ + I_2_ ↔ I_3_^−^). This is confirmed by the CV curve of an aqueous Zn‐ion battery with a Co_3_ZnC/Co/OM‐PC cathode (without sulfur loading), which exhibits a pair of reversible redox peaks ≈1.20 V (Figure S12, Supporting Information).^[^ ^49^, ^50^ ^]^ Two overlapping anodic peaks at ≈0.38 and 0.17 V in the S@Co_3_ZnC/Co/OM‐PC cathode are attributed to the reduction of elemental sulfur to higher‐order polysulfides (Peak II) and the subsequent formation of insoluble sulfides (Peak III), respectively. These peaks indicate multiple reaction mechanisms in Zn‐S batteries. The three overlapping anodic peaks—peak IV (≈1.26 V), peak V (1.34 V), and peak VI (1.42 V)—correspond to the stepwise oxidation of ZnS. First, peak IV is attributed to the oxidation of ZnS to long‐chain polysulfides which convert into short‐chain polysulfides, subsequently. Conversion of long‐chain polysulfides to short‐chain polysulfides (peak V). Finally, peak VI signifies the further oxidation of short‐chain polysulfides to elemental sulfur. Compared to S@OM‐PC and S@PC (Figure S11b,c, Supporting Information), the S@Co_3_ZnC/Co/OM‐PC electrode exhibits lower polarization between the cathodic and anodic peaks, and a higher peak current, demonstrating enhanced reaction efficiency and kinetics. The redox peaks of S@Co_3_ZnC/Co/OM‐PC cathode show symmetry during the cathodic and anodic scans, implying stable and reversible sulfur conversion electrochemistry.

Electrochemical impedance spectroscopy (EIS) further probed the electrochemical interfacial properties. As depicted in **Figure** 7b, the Nyquist plots consist of a depressed semicircle in the high‐ and middle‐frequency regions and a short straight line in the low‐frequency region. The S@Co_3_ZnC/Co/OM‐PC electrode exhibits a smaller semicircle diameter in the medium‐frequency region compared to the S@OM‐PC and S@PC electrodes, indicating lower R_ct_ and enhanced charge‐transfer kinetics. These results are consistent with the GITT analysis discussed above, demonstrating that the 3D interconnected hierarchical porous framework significantly enhances Zn^2^⁺ diffusion kinetics. Both the S@Co_3_ZnC/Co/OM‐PC and S@OM‐PC cathodes are primarily governed by charge transfer processes. As shown in GITT profiles (**Figure** 5e–g), the interfacial reaction resistances are demonstrated by the dip depth in the discharging and charging curves. These dip depth in the discharging and charging profiles indicate the internal resistance to nucleation and activation of ZnS, which can quantify polarization during the charge‐discharge process.^[^ ^51^ ^]^ The S@ Co_3_ZnC/Co/OM‐PC cathode exhibits smaller ΔR_internal_ values between the ZnS nucleation and activation points compared to S@OM‐PC, indicating lower internal resistance. All these results cooperatively identify the significantly boosted sulfur redox kinetics by Co_3_ZnC/Co/OM‐PC.

The electrochemical performance of the prepared sulfur cathode in the aqueous Zn‐S battery was evaluated using galvanostatic charge‐discharge profiles at 0.2 A g^−1^. As shown in **Figure** 7c and S@PC delivers ∼692.6 mA h g^−1^ with a low sulfur utilization of ca 41%, and a high polarization potential of 0.9 V. The electrochemical performance improves with the 3D interconnected hierarchical porous framework in S@OM‐PC, which achieves a capacity of ≈985.5 mA h g^−1^ with a higher sulfur utilization of ≈59% and a relatively lower polarization potential of 0.87 V. By introducing Co_3_ZnC/Co catalyst, the S@Co_3_ZnC/Co/OM‐PC cathode demonstrates significantly enhanced electrochemical performance, achieving a high discharge capacity of ≈1685 mA h g−^1^. This value includes a minor contribution (≈115 mA h g^−1^) from the I_3_^−^/I^−^ redox couple and the carbon host (without loading S), as shown in Figure S13 (Supporting Information). Thus, the sulfur contributes ≈1570 mA hg^−1^ in S@Co_3_ZnC/Co/OM‐PC cathode electrode, corresponding to an impressive value of ≈94% sulfur utilization. Additionally, the S@Co_3_ZnC/Co/OM‐PC electrode exhibits a low polarization potential of 0.67 V, further highlighting its superior electrochemical properties. As shown in Figure S14 (Supporting Information), the S@Co_3_ZnC/Co/OM‐PC and S@OM‐PC electrodes exhibit significantly greater stability than the S@PC electrode during the initial charge‐discharge cycles. This is attributed to the excellent electrolyte permeability enabled by the interconnected open macropores, which shorten mass diffusion paths, and to the presence of a catalyst. To further investigate the electrochemical performance of S@Co_3_ZnC/Co/OM‐PC in an aqueous Zn‐S battery, its rate capability and cycling stability were tested. As shown in **Figure** 7d,e and Figure S15 (Supporting Information), the rate capability of cells with S@Co_3_ZnC/Co/OM‐PC cathode delivers the highest reversible capacity at various current densities compared to S@OM‐PC and S@PC, delivering capacities of ≈1685, ≈1494, ≈1331, ≈1198 and ≈1035 mA h g^−1^ at current density of 0.2, 0.5, 1, 2 and 3 A g^−1^, respectively. When the current density is restored to 0.5 A g^−1^, the capacity recovers to ≈1357 mA h g^−1^ (ca 90.8% of the initial capacity of 1494 mA h g^−1^ at 0.5 A g^−1^). In contrast, S@OM‐PC and S@PC retain only ≈80% and 75% (**Figure** 7d), respectively. These results demonstrate the accelerated polysulfides reaction kinetics in the S@Co_3_ZnC/Co/OM‐PC cathode.

#### 2.4.2 Reaction Pathways of Sulfur Conversion

**Figure 8:**
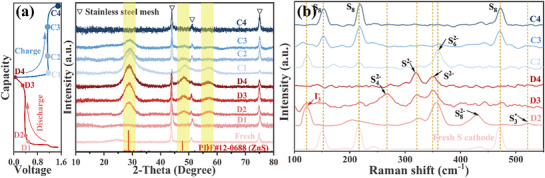
The GCD profile of S@Co_3_ZnC/Co/OM‐PC at 0.2A g^−1^ (left) and Ex situ XRD patterns (right); b) ex situ Raman spectra during discharge and charge.

To unveil the conversion mechanism of the sulfur cathode in the hybrid electrolyte‐based Zn‐S cell, we first examined *ex situ* XRD and Raman analysis of the S cathode at different charging/discharging states. As shown in **Figure** **8**a, the pristine cathode material shows only diffractions from the stainless‐steel mesh, resulting from the poor crystallinity of sulfur. When S@Co_3_ZnC/Co/OM‐PC is discharged to point D2, the diffraction peaks at ≈28.6°, 47.6°, and 56.3° show up, which correspond to the ZnS structure, demonstrating part of the S has been converted into ZnS. Subsequently, with deeper discharge, S species continue to be converted into ZnS. The discharge process demonstrates that S undergoes a conversion reaction from S to ZnS. In the charge process, it can be observed that the diffraction peaks of ZnS gradually weaken and finally disappear at the fully charged 1.5 V (C4), indicating the reversible conversion from ZnS to the amorphous structure of S. Furthermore, the sulfur‐containing species formation in the S@Co_3_ZnC/Co/OM‐PC electrode during the charge and discharge process was detected by ex situ Raman. As shown in **Figure** 8a, only the S peaks at 151 cm^−1^, 216 cm^−1,^ and 469 cm^−1^ of the pristine S@Co_3_ZnC/Co/OM‐PC electrode were observed.^[^ ^51^ ^]^ Upon discharging to the point D2, the peaks at ∼358 and 429 cm^−1^ appeared which can be attributed to S_6_^2−^ and S_8_^2−^ species,^[^ ^52^, ^53^ ^]^ respectively, meanwhile, the peaks of S disappeared. The peak at 521 cm^−1^ can be assigned to S_3_^*−^, from the dissociation of S_6_^−^ to S_3_^*−^ radical.^[^ ^54^ ^]^ Further discharge to point D3, the formation of ZnS had already been demonstrated by the signals at ≈321 and 351 cm^−1^, and S_4_^2−^ can be detected at 268 cm^−1^.^[^ ^51^, ^53^ ^]^ When the battery is quantitatively discharged, the peaks of polysulfides disappear, only ZnS signals can be observed, meaning that almost all the S species have been transformed into ZnS. During the charging process, the polysulfides signals reappear in a reversed sequence relative to the discharge process. Upon battery discharging to point C2, the peaks of ZnS disappeared and the signals of S_6_^2−^ species were detected, clearly indicating that the ZnS has been converted into various polysulfides.^[^ ^52^ ^]^ Further charge to point C3, S_6_^2−^ species and S both can be detected, meaning that parts of the polysulfides have been transformed into S. When the cell is charged deeply, the signals of polysulfides disappear, only S peaks can be detected at the end of charging, indicating most of polysulfides have been transformed into elemental S. The peak of I_3_^−^ at ≈122 cm^−1^, which can be detected in the S cathodes during both the discharge and charge process, shows the reversible transition between I_3_^−^ and I^−^ (**Figure** 8i), proving the existence of I_3_^−^/ I^−^ redox couple during the sulfur conversion, consistent with the CV result (**Figure** 7a; Figure S12, Supporting Information).^[^ ^55^, ^56^ ^]^ The I_3_^−^ plays an important role in the sulfur conversion process. Because the I_3_^−^ can be well associated with S_8_ and ZnS to form S_8_⋅I_3_^−^ and ZnS⋅I_3_^−^ species, respectively.^[^ ^46^, ^57^ ^]^ The redox reactions of sulfur are facilitated by the association effect with I_3_^−^.^[^ ^58^ ^]^

#### 2.4.3 Electrochemical Stability Mechanism

**Figure 9:**
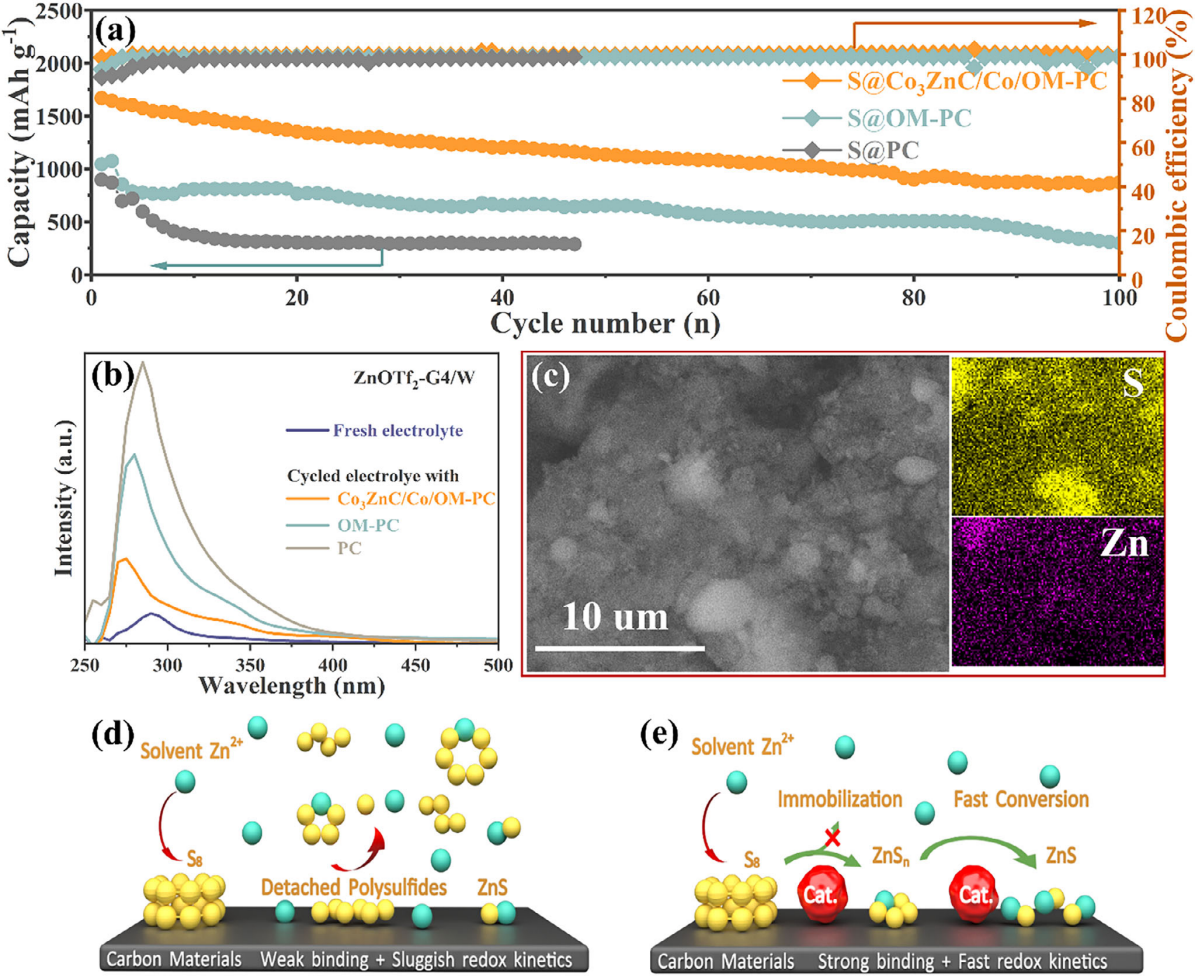
a) The cycling stability of S@OM‐Co_3_ZnC/Co/PC, S@OM‐PC and S@PC at 0.5 A g^−1^; b) The UV–vis spectra of cycled electrolytes cycled in different cells with different cathodes at 0.05 Ag^−1^ for 3 cycles using an electrolyte without ZnI_2_. c) the elements maps of the post‐cycling electrode. The schematics diagram of the S conversion on the carbon host without d) the catalyst and e) with the catalyst.

As shown in **Figure** **9**a, compared with S@OM‐PC and S@PC cathodes, S@Co_3_ZnC/Co/OM‐PC cathode retains a higher capacity at a current density of 0.5 A g^−1^ after 100 cycles, indicating that S@Co_3_ZnC/Co/OM‐PC cathode has good cycling stability. The cells with S@Co_3_ZnC/Co/OM‐PC cathode achieved a very high Coulombic efficiency (CE) of ca 99.7, whereas the cells with S@OM‐PC and S@PC electrodes exhibit a lower CE of ≈98.6% and ≈97.3%, respectively (**Figure** 9a), resulting in weak absorbability of OM‐PC and PC toward polysulfides. Compared with the separators from the cells based on S@PC, S@OM‐PC, and S@Co_3_ZnC/Co/OM‐PC after 50 cycles (Figure S16, Supporting Information), it shows that the diffusion of polysulfide intermediates is considerably more pronounced in S@PC and S@OM‐PC cathodes than in those with the catalyst‐integrated cathode S@Co_3_ZnC/Co/OM‐PC. Accordingly, the electrolyte in the S@Co_3_ZnC/Co/OM‐PC cell showed only a slight yellowish color after 3 cycles at 0.05 A g−^1^ as compared with S@OM‐PC and S@PC (Figure S17, Supporting Information). The UV–vis spectra of the cycled electrolytes strongly support the effective suppression of the polysulfide shuttle effect in S@Co_3_ZnC/Co/OM‐PC cell (**Figure** 9b). This can be attributed to the strong chemical adsorption and enhanced conversion kinetics facilitated by the Co_3_ZnC/Co electrocatalyst.^[^ ^59^ ^]^ The cells with the S@Co_3_ZnC/Co/OM‐PC cathode demonstrated excellent cycling stability with a minimal capacity decay rate of only ≈0.40% per cycle at 1 A g^−1^ and ≈0.37% per cycle at 2 A g^−1^ over 100 cycles, respectively (Figure S18, Supporting Information). To further explore the stability of the S@Co_3_ZnC/Co/OM‐PC, the electrode after lasting long charge‐discharge cycles was investigated by SEM, EDS, and XPS. As shown in Figure S19 (Supporting Information), the morphology of S@Co_3_ZnC/Co/OM‐PC remained unchanged after undergoing 50 long cycles (at 0.5 A g^−1^), demonstrating that S@Co_3_ZnC/Co/OM‐PC possesses a stable structure that can withstand volume expansion between S and ZnS during the long cycle testing. The EDS element spectra (Figure S20a,b, Supporting Information) show that S@Co_3_ZnC/Co/OM‐PC has a high S retention capability during the long charge‐discharge cycle testing and the elemental mapping (Figure 9c; Figure S20c, Supporting Information) confirms that S was still uniformly distributed in the host material, suggesting the strong adsorption of sulfur species on Co_3_ZnC/Co/OM‐PC. The C 1s peaks (Figure S21, Supporting Information) of Co_3_ZnC/Co/OM‐PC electrode after 50 cycles show that the metal‐carbon (M‐C) bond derived from the Co_3_ZnC at 283.7 eV was still present and has not weakened at all, suggesting a very good stability of Co_3_ZnC catalyst during the charging and discharging process. Thus, Co_3_ZnC/Co/OM‐PC exhibits strong stability and can trap polysulfide intermediates successfully and effectively.

The S@Co_3_ZnC/Co/OM‐PC cathode delivers an initial capacity of ≈1151 mA h g^−1^ at a high current density of 3 A g^−1^ (Figure S22, Supporting Information), maintains a stable capacity of ≈722 mA h g^−1^ up to 110^th^ cycle. However, when the cell is opened, the electrolyte evaporates rapidly, causing the capacity to drop sharply to ≈322 mA h g−^1^. After replacing the Zn anode, separator, and adding fresh electrolyte, the capacity of S@Co_3_ZnC/Co/OM‐PC cell recovered to ≈838 mA h g^−1^. However, after 204 cycles, a dendrite‐induced short circuit caused the capacity to drop dramatically to ≈299 mA h g−^1^. Subsequently, replacing the Zn anode once more allowed the capacity to recover to ≈1074 mA h g−^1^. These results indicate that the capacity fading in S@Co_3_ZnC/Co/OM‐PC is mainly related to electrolyte decomposition (Figure S23, Supporting Information), which leads to cell expansion through O_2_ and H_2_ generation and subsequent water loss. Severe Zn dendrites growth (Figure S24, Supporting Information) can pierce the separator as demonstrated by the XRD analysis (Figure S25, Supporting Information) of the separator, leading to short circuits (Figure S26, Supporting Information). This is a critical factor that leads to reduced cycle efficiency. As shown in Figure S22 (Supporting Information), the S@Co_3_ZnC/Co/OM‐PC cathode exhibits an extremely low‐capacity decay of only ≈0.027% per cycle during 400 cycles at 3 A g^−1^. Notably, after replacing the Zn anode following a short‐circuit event or replenishing the electrolyte when the cell expanded, the electrode significantly restored its performance, comparatively better than recently published reports (Table S2, Supporting Information). These results collectively demonstrate that the Co_3_ZnC/Co catalysts markedly enhance cycling stability.

A schematics diagram in **Figure** 9d–e represents a mechanism of S conversion on the carbon host without and with Co_3_ZnC catalyst. The sluggish kinetics of the polysulfide conversion is usually observed for porous carbon matrices loaded with sulfur/polysulfides, attributed to the intrinsic inertness of carbon and its weak interaction with polysulfides. The metal carbide catalyst integrated in cathode, however, exhibits stronger chemical adsorption for polysulfides. This can be attributed to the sulfophilic surface moieties and higher electronic conductivity of the cementite‐like carbides. These factors are supposed to effectively facilitate the polysulfide conversion through enhanced charge transfer kinetics.^[^ ^60^ ^]^ The transition metal atom sites of the carbide material provide a high catalytic activity for the sulfur conversion.^[^ ^61^ ^]^ Metal carbides as catalysts are widely used in Li‐S batteries to alleviate the shuttle effect and promote overall performance. Recent studies also showed that the bimetallic carbides such as Co_3_ZnC exhibit strong chemical adsorption for polysulfides. However, the Sabatier principle, as a fundamental concept of heterogeneous catalysis, states that only moderate adsorption can achieve an optimum conversion of metal polysulfides.^[^ ^62^ ^]^ Rationally adjusting the valence states of the coordinatively unsaturated active metal centers can tailor the electronic properties, the adsorption capacity, and catalytic activity of the electrocatalysts.^[^ ^63^ ^]^ The redox‐active Co^2^⁺/Co⁰ pair may form chemical bonds with intermediate polysulfides via Lewis acidic Co^2^⁺ sites, while the electron transfer between Co⁰ and Co^2^⁺ synergistically enhances electrocatalytic activity, significantly improving overall catalytic performance.^[^ ^33^, ^35^ ^]^ On the other hand, the incorporated Zn^2+^ can effectively modulate the balance of the predominantly active of Co^2+^/Co^0^ pair, which leads to inhibited shuttle effect and accelerates the catalytic conversion kinetics of polysulfide intermediates.^[^ ^35^ ^]^ These factors together may constitute at least a conceptual explanation why the reported S@Co_3_ZnC/Co/OM‐PC cathode shows high conversion kinetics of polysulfides, and good cycling stability. Moreover, the 3D interconnected hierarchical porous carbon matrix facilitates efficient electrolyte penetration and ion diffusion, while providing a high density of accessible electroactive sites.^[^ ^24^, ^25^ ^]^ These features synergically enable rapid confinement of polysulfides and significantly accelerate the redox kinetics of zinc polysulfide species.

Finally, the influence of sulfur mass loading on the discharge capacity of S@OM‐Co_3_ZnC/Co/PC (Figure S27, Supporting Information) was investigated. The Co_3_ZnC/Co/PC cathodes exhibited capacities of ≈1688, ≈1657, ≈1066, ≈1094, and ≈1191 mA h g^−1^ with a S loading of 1.1, 2.0, 3.5, 4.1, and 5.8 mg cm^−1^, respectively. The polarization voltage between charge and discharge plateau slightly increased, however, the capacity remained acceptably high with increased S loading, which is critical to develop high mass loading cathodes for practical applications. As shown in Figure S28 (Supporting Information), the S@OM‐Co_3_ZnC/Co/PC cathode with high areal sulfur loading of 4.1 and 3.5 mg cm^−2^ shows a good initial capacity of ≈992 and 1088 mA h g^−1^ with a high CE, respectively. The reversible capacity of the electrode with an S loading of 3.5 mg cm^−2^ was maintained with a capacity of ≈533 mA h g^−1^ over 100 cycles at 0.2 A g^−1^, whereas 591.8 mA h g^−1^for the electrode with a S loading of 4.1 mg cm^−2^ over 50 cycles at 0.2 A g^−1^.

## 3 Conclusion

We designed and synthesized a hierarchically ordered macro‐mesoporous carbon‐supported Co_3_ZnC/Co hybrid catalyst material, derived from a hierarchically ordered macro‐mesoporous, Co‐doped Zn‐ZIF via in situ growth of ZIF‐8 within the interstitial spaces of a self‐assembled polystyrene monolith, followed by the removal of polystyrene nanoparticles. Systematic experimental investigations demonstrate that the 3D hierarchically ordered macro‐meso‐microporous architecture facilitates Zn^2^⁺ diffusion and enhances reaction kinetics during the charge‐discharge process. The Co_3_ZnC/Co catalyst not only improves the weak surface affinity of the porous carbon host for polysulfide trapping but also accelerates the redox conversion of polysulfides. This significantly enhances sulfur utilization, mitigates the shuttle effect, and improves cycling stability. Compared with the recent publications, the aqueous Zn‐S battery assembled with the S@Co_3_ZnC/Co/OM‐PC electrode delivered a synergistic high discharge capacity of ≈1685 mA h g^−1^ (including the contribution of ≈115 mA h g^−1^ from I_3_^−^/I^−^ redox couple and the carbon host without S loading) at 0.2 C and exhibits ≈0.027% capacity decay per cycle after 400 cycles at 3 A g^−1^. Even at a high sulfur loading of 3.5 mg cm^−2^, the S@Co_3_ZnC/Co/OM‐PC battery achieves an initial capacity of ≈992 mA h g^−1^ with a high coulombic efficiency of above 99% and retains a capacity of ≈533 mA h g^−1^ over 100 cycles at 0.2 A g^−1^. Different from the mainly reported mechanism of solid‐solid conversion of sulfur in aqueous Zn‐S battery, this work reveals that S cathode undergoes intermediate polysulfides conversion, finally transforming into ZnS. This work provides mechanistic insights and a versatile design to prepare hierarchical MOF‐derived catalyst/OM‐PC heterostructures with suitable polysulfide adsorption and high electrocatalytic activity for high‐performance aqueous Zn‐S batteries.

## 4 Experimental Section

Detailed procedures for synthesis and characterization are provided in Supplementary Information.

## Conflict of Interest

The authors declare no conflict of interest

## Supporting information



Supporting Information

## Data Availability

The data that support the findings of this study are available from the corresponding author upon reasonable request.
