# Supplementary material for: Cobalt‐Based Catalyst Integration Into a Hierarchically Ordered Macro‐Meso‐microporous Carbon Cathode for High‐performance Aqueous Zn‐Sulfur Batteries
Source: Adv Sci (Weinh). 2025 Aug 21;12(43):e09945. doi: 10.1002/advs.202509945 (PMC12631863; doi:10.1002/advs.202509945)
Supplement: Supplementary file 1 — Supporting Information [file ADVS-12-e09945-s001.docx]

Support Information

Cobalt-based Catalyst integration into a hierarchically ordered macro-meso-micro porous carbon cathode for high-performance aqueous Zn-Sulfur batteries

Liangzhen Liu,^a^ Mian Zahid Hussain^a*^, Da Lei^a^, Olivier Henrotte^b^, Emiliano Cortés^b^, Aliaksandr S. Bandarenka^c*^, and Roland A. Fischer ^a*^

^a^ Chair of Inorganic and Metal-Organic Chemistry, Catalysis Research Center, Department of Chemistry, School of Natural Sciences, Technical University of Munich, Garching 85748, Germany

^b^ Nanoinstitute Munich, Faculty of Physics, Ludwig-Maximilians-Universität München (LMU), 80539 Munich, Germany

^c^ Physics of Energy Conversion and Storage, Department of Physics, Catalysis Research Center, TUM School of Natural Sciences, Technical University of Munich, Garching 85748, Germany

The authors have declared that no conflict of interest exists

*Corresponding authors:

E-mail addresses: [roland.fischer@tum.de](mailto:roland.fischer@tum.de) (R.A. Fischer), [zahid.hussain@tum.de](mailto:zahid.hussain@tum.de) (M.Z. Hussain), [bandarenka@ph.tum.de](mailto:bandarenka@ph.tum.de) (A.S. Bandarenka)

**Experimental Section**

***Chemicals.*** Potassium persulfate (K_2_S_2_O_8_, 99 wt.%), Styrene (99%), Polyvinyl pyrrolidone (PVP, 30K, Sigma Aldrich), Zinc nitrate hexahydrate (Zn(NO_3_)_2_∙6H_2_O, 99%,), 2-methylimidazole (98%), tetrahydrofuran (99%), N,N-dimethylformamide (99.5%), Carboxmethyl Cellulose (CMC, Nanografi Nano Technology), Zinc trifluoromethanesulfonate (Zn(OTf)_2_, 98%), Zinc iodide (98%), Tetraglyme (99%), Zn foil (99.9%), Super 65, stainless steel mesh.

All chemicals and reagents were bought from Sigma-Aldrich and used directly without further purification. Ultrapure water (Millipore Milli-Q grade) with a resistivity of 18.2 MΩ was used in all experiments.

***Synthesis of*** ***Co:OM-Zn-ZIF-8(Zn), OM-ZIF-8 and ZIF-8.*** Zn(NO_3_)_2_·6H_2_O (8.15g, 27.4 mmol), Co(NO_3_)_2_·6H_2_O (360 mg, 1.24 mmol) and of 2-methylimidazole (6.75 g, 85.2 mmol) were dissolved into 45 mL of methanol solution. The ordered PS template (d = 260 nm) was immersed into this solution for 4 hours, and further treated with a vacuum degassing for 12 min. After that, this impregnated PS template was taken out and dried at 50 °C overnight and then was transferred to immerse into a 50 mL mix solution of methanol and ammonia solution (3:2, v/v) for 24h to trigger the formation of ZIFs. The PS template was removed by THF.

***Fabrication of Co_3_ZnC/Co/OM-PC, OM-PC and PC.*** The Co_3_ZnC/Co/OM-PC, OM-PC and PC was obtained by a calcination treatment of Co:OM-Zn-ZIF-8(Zn), OM-ZIF-8 and ZIF-8 at 900 °C under inert gas with Ar flow for 2 h in a tube furnace with a heating rate of 5 °C min^-1^, respectively.

***Preparation of sulfur cathode.*** *Samples of* 50 wt.% sublimed sulfur and 50 wt.% host materials (Co_3_ZnC/Co/OM-PC, OM-PC, PC) were mixed and heated in an autoclave at 155℃ for 12 h. The resultant mixture was dispersed with Super 65 and Carboxymethyl cellulose (CMC) in DI-water at a weight ratio of 8:1:1, and the slurry obtained was coated on stainless steel mesh and dried at 70℃ for 12 h. The areal sulfur loading of the cathode was 1.5-2.5 mg cm^−2^ for routine electrochemical measurements.

***Preparation of electrolytes.*** The preparation of the homogenous electrolyte solution 2 mol Zn(OTf)_2_/ZnI_2_/G4/W was carried out by adding Tetraglyme (G4) into water (volume ratio 2:3), and then dropping Zn(OTf)_2_ salt into the above solution with a constant concentration of 2 mol, of which ZnI_2_ was added as an additive (accounting for 1 wt. % of the mass of Zn(OTf)_2_ salt).

**Preparation of polysulfide (ZnS_x_).** The ZnS_x_ solution was prepared by reacting to the solid ZnS with sulfur powders. In a typical process, 96.2 mg, 3mmol of sulfur and 97.5 mg, 1mmol ZnS powders were dispersed into 10 mL of water, the mixed solution of G4 and Water (Tetraglyme: water = 2:3), and G4 in a 50 mL Teflon-lined stainless-steel autoclave, respectively. The suspension was sonicated for 30 min, then left for reaction at 180℃ for 12h. As shown in Figure S1, the color of the solution doesn’t change with pure water, and the UV-Vis spectra shows that there isn’t any polysulfides formation. However, the color of the solution turned yellow with the mixed solution of G4 and Water, whereas the color turned brown in the G4 solution. The UV-Vis spectroscopy results indicate that S_4_^2-^ and S_6_^2-^ were generated in mixed solution and G4.


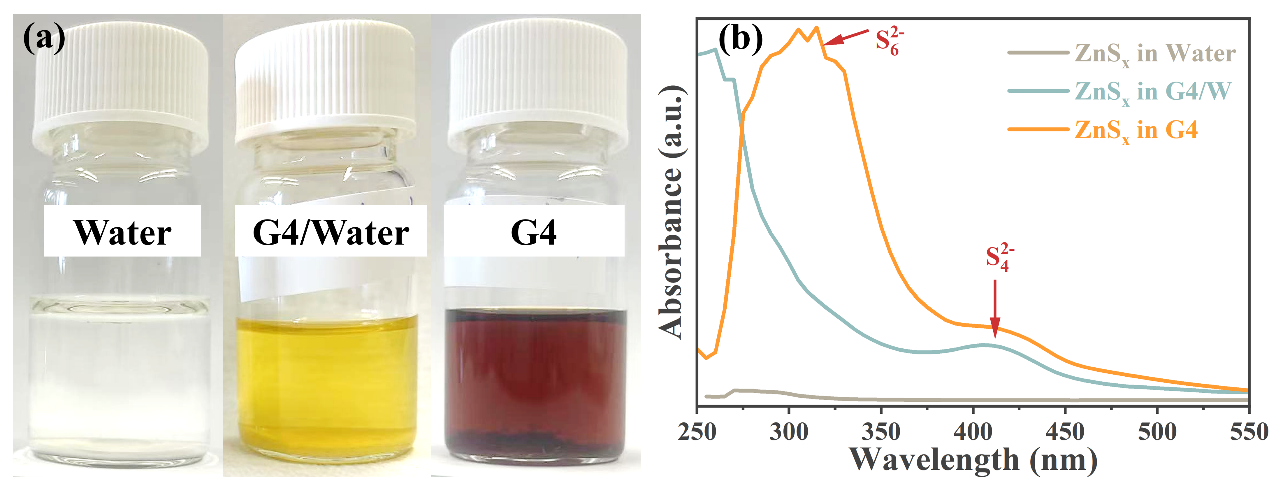


**Figure S1 (a)** Digital images and (b) UV-vis spectra of ZnS_x_ synthesized in water, the mixed solution and G4 solvent.

Battery assembly and performance measurements.

The zinc sulfur batteries, as shown in **Fig. S1a**, were tested using assembled CR2032 coin cells (**Fig. S1b**) to test the electrochemical performance of different sulfur cathode electrodes. The sulfur cathode was prepared by mixing S@Co_3_ZnC/Co/OM-PC, S@OM-PC and S@PC (80 wt.%), with conductive carbon (10 wt.%) and Carboxymethyl Cellulose Binder (CMC) (10 wt.%) in deionized water. For CR2032 coin cells, the mixed slurry was evenly coated on the stainless-steel mesh with a diameter of 12 mm, and the loading mass of sulfur was unified between 2.0-3.5 mg cm^-2^. All CR2032 coin cells use 0.1 mm thick zinc foil as anode, glass fiber membrane as separator and 2M Zn(OTf)_2_/G4/ZnI_2_/water were used as electrolytes. All batteries in this work were rested for 12 h before testing. Electrochemical impedance spectroscopy (EIS) tests were conducted in the frequency range of 10^-2^-10^5^ Hz under open-circuit voltage. Cyclic Voltammetry (CV) was tested in the range of 0.05 to 1.5 V with a scan rate of 0.1 mV s^-1^. The test equipment for CV and Electrochemical Impedance Spectroscopy (EIS) was an Auto lab electrochemical workstation. The rate and cycle performance of the batteries were measured by the Neware CT-4008T battery test system at room temperature. The batteries underwent two pre-cycles at 0.2 A g^-1^ before cycling at 0.5-3 A g^-1^.


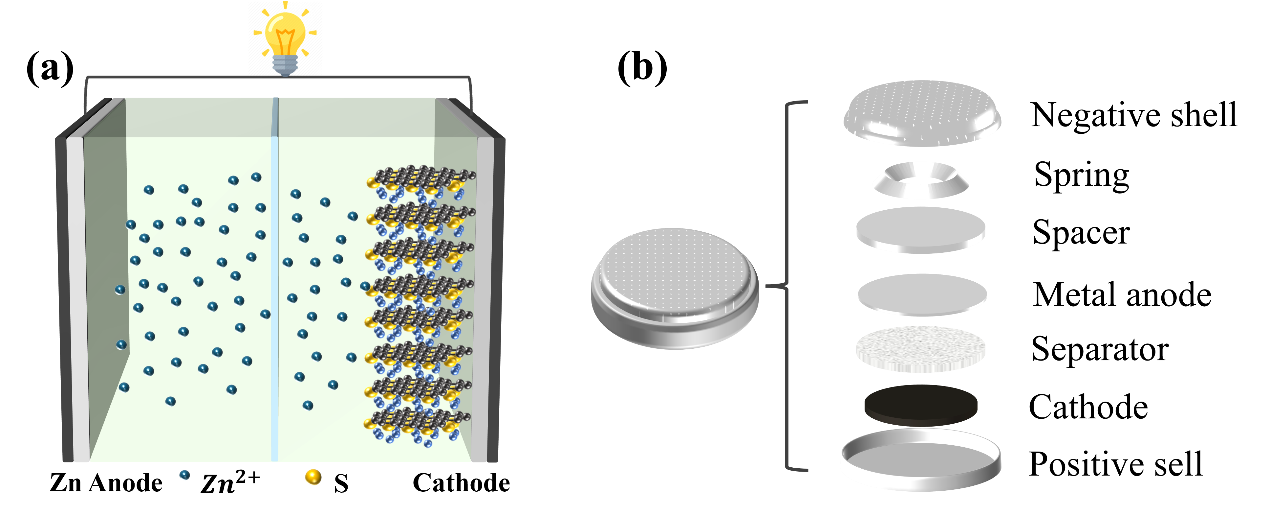


**Figure S2** (a) The diagram of the aqueous zinc-sulfur batteries. (b) the Schematic diagram of button cell structure.

The Symmetric batteries were tested (identical electrodes) with ZnS_x_-containing electrolyte. The cyclic voltammetry (CV) was performed in the potential range of -1.2 to 1.2 V with a scan rate of 10 mV s^-1^. The linear sweep voltammetry (LSV) was performed from -1.2 to 1.2 V with a scan rate of 5 mV s^-1^. The Tafel slope is extracted from the linear part of η vs. log|j| plot and calculated as following equation:

$\eta=a+blog |j|$ (Equation S1)

where, b is the Tafel slope (mV. dec.^-1^) intercept related to the exchange current density (j_0_​), and η is the overpotential.

Activation energy (E_a_) was calculated from temperature-dependent EIS measurements at five different temperatures (10, 20, 30, 40, and 50 °C), by analysing temperature-dependent charge-transfer resistance (R_ct​_) using the **Arrhenius equation:**

$\frac{1}{R_{ct}}\propto k=A.e^{\frac{-E_{a}}{RT}}$ (Equation S2)

Where, k is the rate constant (*k*); *R* presents universal gas constant (8.314 J. mol^-1^·K^-1^); *T* means temperature (K); *n* is the number of electrons transferred; *F* = Faraday’s constant (96,485 C.mol^-1^).

**Galvanostatic Intermittent Titration Technique (GITT) measurement.**

The GITT measurement was performed at the current density of 0.1 C for 15 min and the rest for 30 min and repeat this cycle until the discharge voltage is less than or equal to 0.05, or the charge voltage is bigger than or equal to 1.5V. In the GITT measurement, as shown in **Figure S2a and c**, the total overpotential ($\text{η}$) can be calculated by the difference between the measured cell voltage during the current pulse and the voltage at the end of the relaxation period, as shown following:

$\text{η}\text{ }\text{=}\text{ }\text{|}\text{E}_{\text{meas}}\text{- }\boldsymbol{E}_{\text{eq}}\text{| = }\text{|CCV-QOCV}\text{|}$ (Equation S3)

The polarization during the electrochemical operation was quantified by introducing the internal resistance, based on the following relation

${\Delta R}_{internal}\left( \Omega\right)=\left| {\Delta V}_{QOCV-CCV} \right|/I_{applied}$ (Equation S4)

where $\text{∆V}$ is the voltage difference between the points of quasi-open circuit voltage and closed-circuit voltage, and 𝐼𝑎𝑝𝑝𝑙𝑖𝑒𝑑 is the current applied.

The chemical diffusion coefficient of zinc ($D_{\mathrm{Zn}^{2+}}$) can be easily calculated based on GITT using the following Equation:

$\text{D}_{\text{Zn}^{\text{2+}}}\text{= }\frac{\text{4}}{\text{π∆t}}\text{(}\frac{\text{m}_{\text{b}}\text{V}_{\text{M}}}{\text{M}_{\text{B}}\text{S}}\text{)}{\text{(}\frac{\text{∆}\text{E}_{\text{S}}}{{\Delta E}_{\text{t}}}\text{)}}^{\text{2}}$ (Equation S5)

Where $m_{B}$ is the electrode active mass, $V_{M}$ and $M_{B}$ represent the molar volume and the molar mass of the electrode materials, respectively. Here, $\frac{\text{V}_{\text{M}}}{\text{M}_{\text{B}}}$ is obtained from the density of the active materials. τ, $m_{B}$, and S are the values of the experimental condition. Here, the geometric area of the electrode was used as S. ${\Delta E}_{s}$ and ${\Delta E}_{\tau}$ are defined as **Figure S3b and d**.


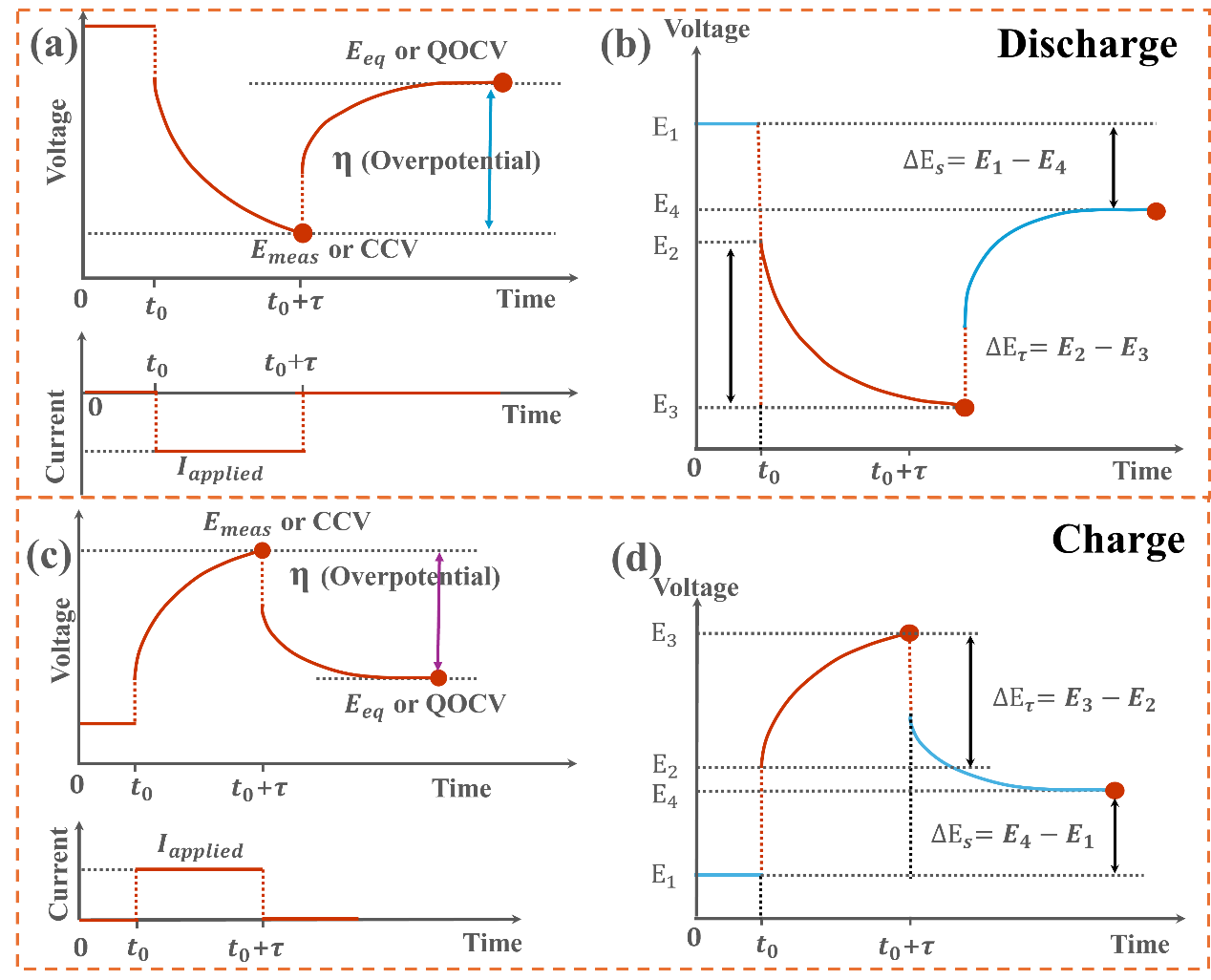


Figure S3 Schematic diagram of the two linear correlation assumptions in one galvanostatic titration step. The diagram represents the definition of the (a and c) overpotential ($\boldsymbol{\eta}_{\boldsymbol{overpotential}}$) and (b and d) $\boldsymbol{\Delta E}_{\boldsymbol{s}}$ and $\boldsymbol{\Delta E}_{\boldsymbol{\tau}}$ during the discharge and charge, respectively.

**Material Characterizations**

The microstructure of the sample was characterized by scanning electron microscope (SEM, JEOL JSM-7500F). XRD patterns were detected using X-ray diffraction (XRD, an X’Pert PRO PAN analytical instrument with a Ni-filtered Cu Kαradiation (λ= 1.5406 Å). The sulfur content of the sample was detected by thermogravimetric analyzer (TG, Labsys Evo, Setaram). Raman spectra were recorded by using a Raman microscope (Renishaw plc (in Via reflex) with 532 nm). Brunauer-Emmett-Teller (BET) method was applied to calculate the specific surface area of the products via N_2_ adsorption-desorption isotherms on Autosorb IQ (Quantachrome) at 77 K.

**Supporting Figures and Tables**


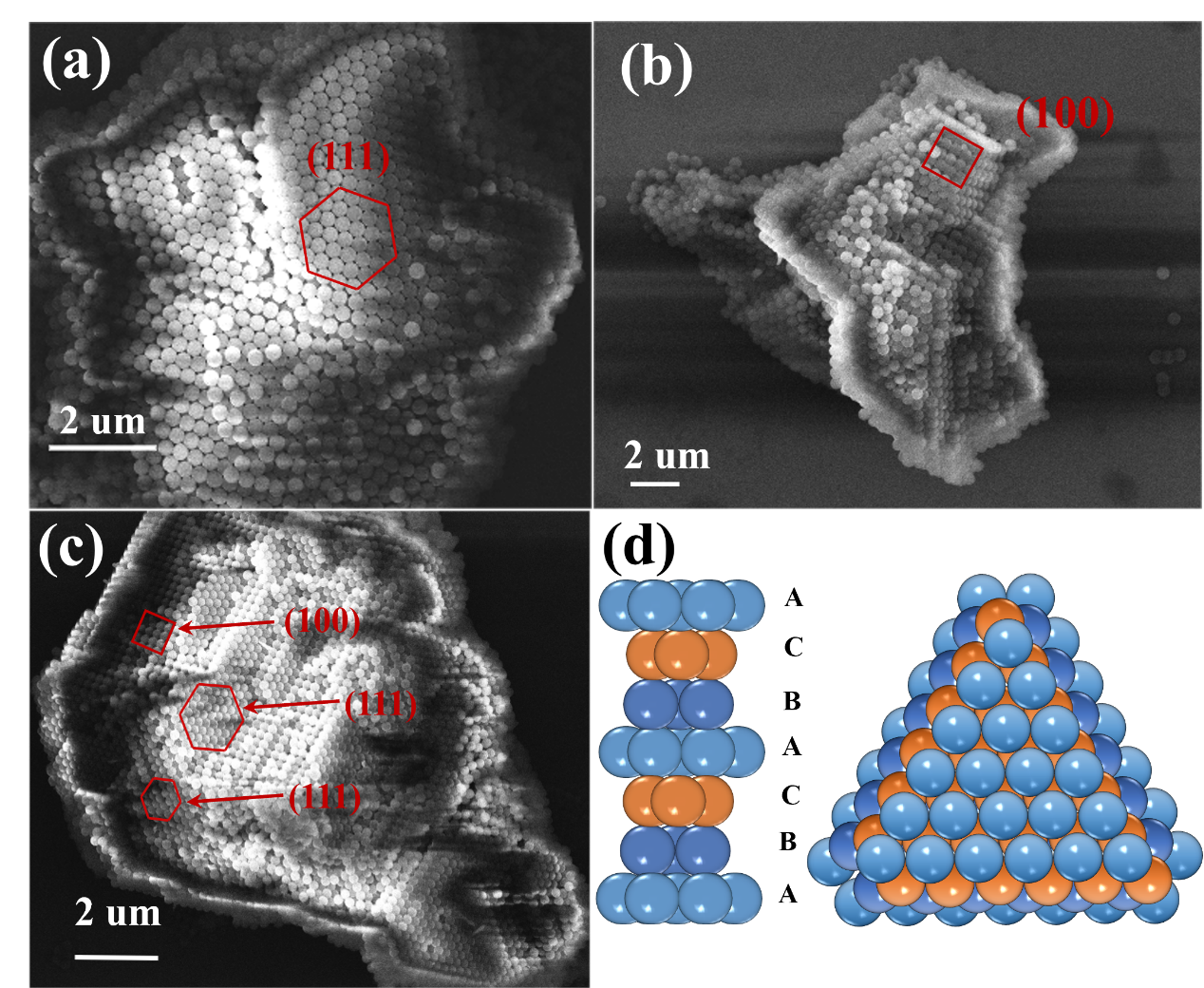


Figure S4a-c SEM image of polystyrene template. d, Schematic diagram of face-centered cubic (fcc) packing.

As shown above, SEM image (Fig. S2a-c) clearly shows that the synthesized polystyrene nanospheres are monodisperse, with a diameter of around 300 nm and a dispersion smaller than 5%, which plays a key role in constructing highly ordered polystyrene opal. A quite uniform color under nature light which transforms from green to pink when observation angle changes can be observed on the polystyrene monolith, which means a good alignment of nanospheres in the formed polystyrene opal. To clearly determine the structure of polystyrene opal attained, the side facets from sample cleaved edges were characterized by SEM. The SEM images of different side facets observed in the cleaved edges are shown in Fig. S2a-b, where square and triangle arrangements of nanospheres are obviously shown, respectively. A triangular arrangement in Fig. S2a corresponds to type plane in fcc structure, while a square arrangement in Fig. S2b corresponds to type plane of fcc structure. A typical terrace view is shown in Fig.S2c, where several different crystalline planes are clearly revealed, which consistent with the fcc crystal structure. This directly confirms that the polystyrene nanospheres self-assembled in an fcc packing structure (Fig.S2d) in this work.^1^


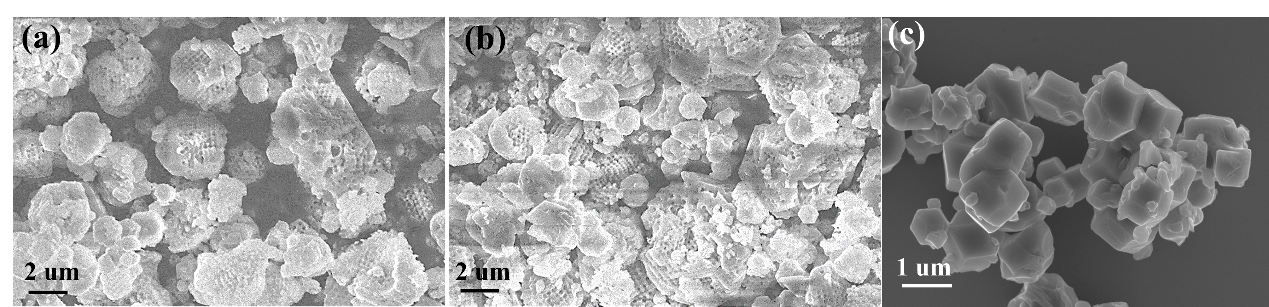


Figure S5 SEM image of (a) Co:OM-ZIF-8(Zn), (b) OM-ZIF-8 and (c) ZIF-8.


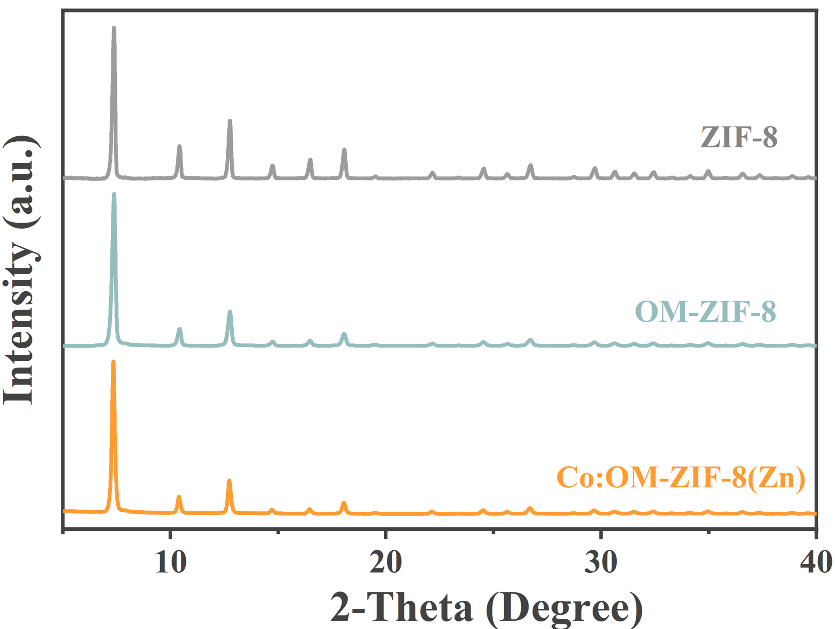


Figure S6 PXRD patterns of Co:OM-ZIF-8(Zn), OM-ZIF-8(Zn) and ZIF-8(Zn).


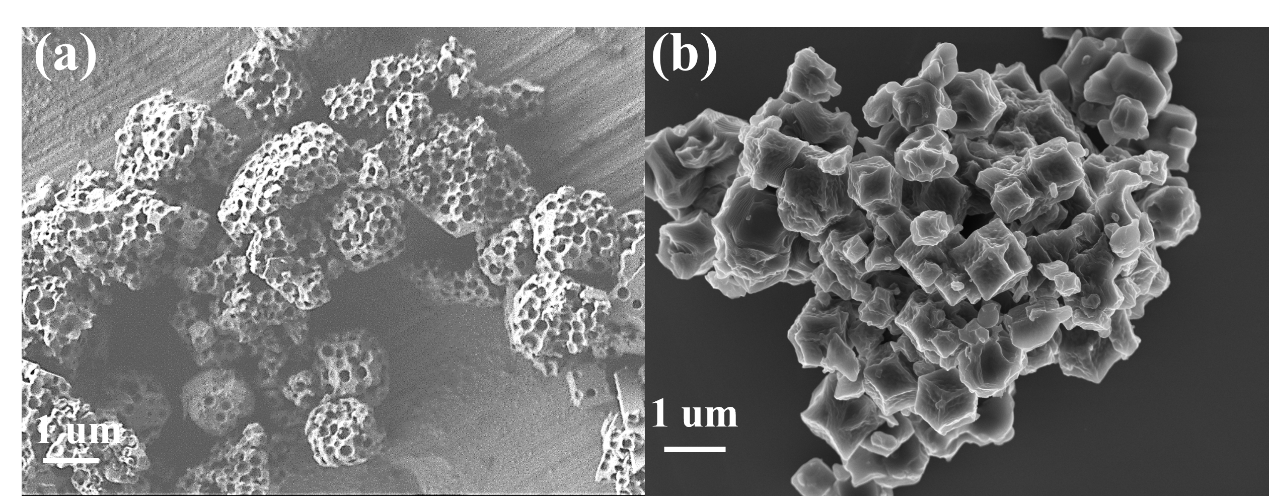


Figure S7 SEM image of (a) OM-PC, and (b) PC.


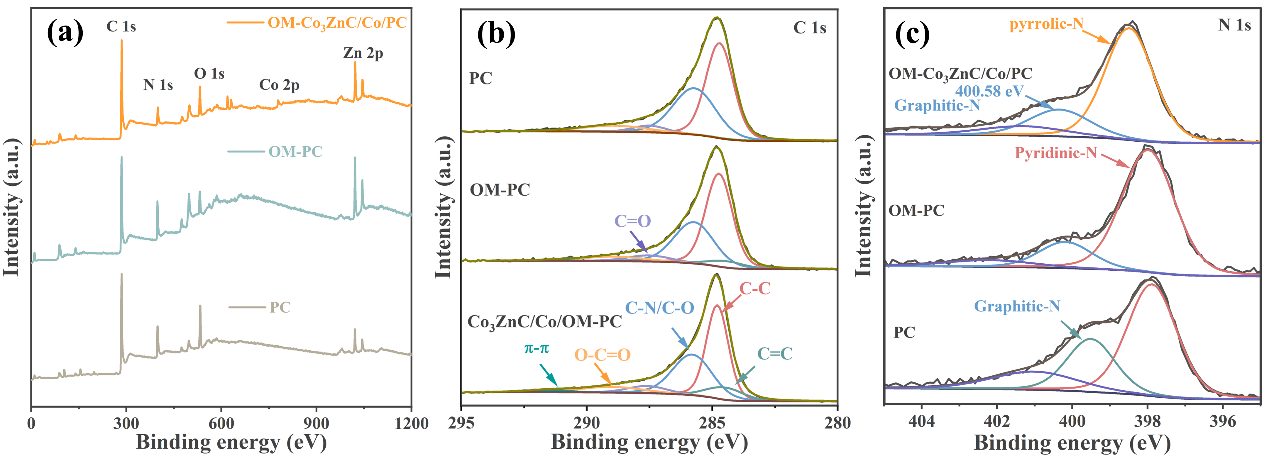


Figure S8 (a) XPS survey spectrums; high-resolution XPS spectra of (b) C 1s and (c) N 1s.


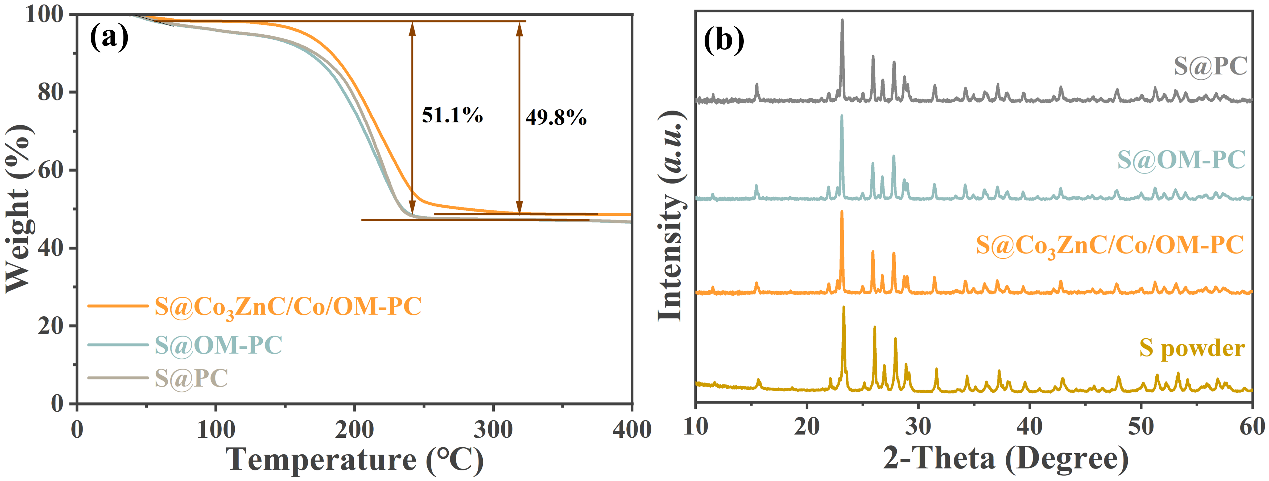


Figure S9 (a) TGA and (b) PXRD patterns of S@Co_3_ZnC/Co/OM-PC, S@OM-PC and S@PC.


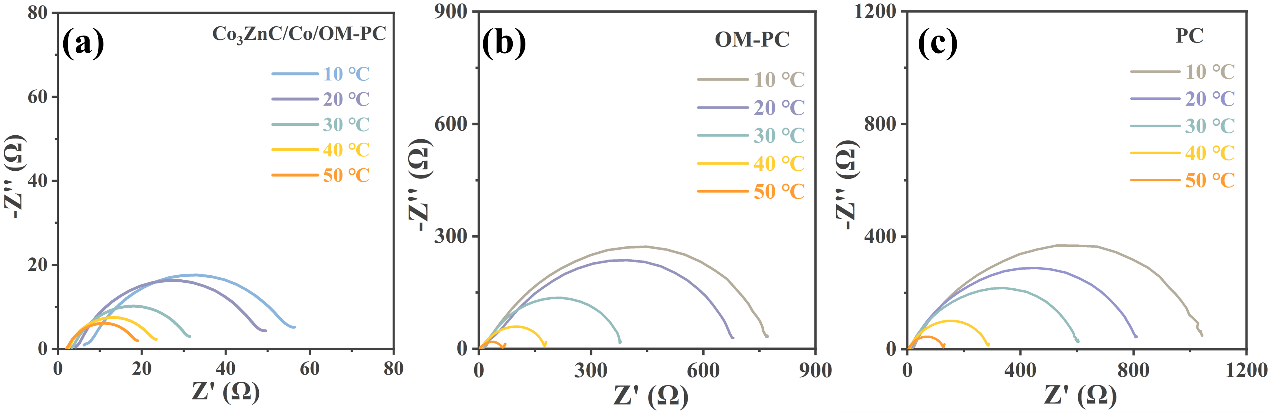


Figure S10 Nyquist plots for Co_3_ZnC/Co/OM-PC, OM-PC and PC symmetric cells at different temperatures from 10℃ to 50℃.


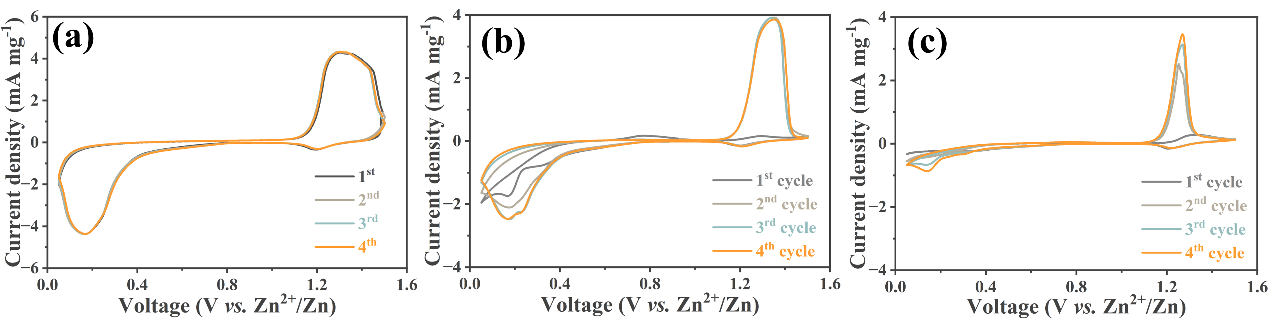


Figure S11 The first four cycles of CV curves of (a) S@Co_3_ZnC/Co/OM-PC, (b) S@OM-PC and (c) S@PC. (S loading: 1.2-2.0 mg cm^-2^)


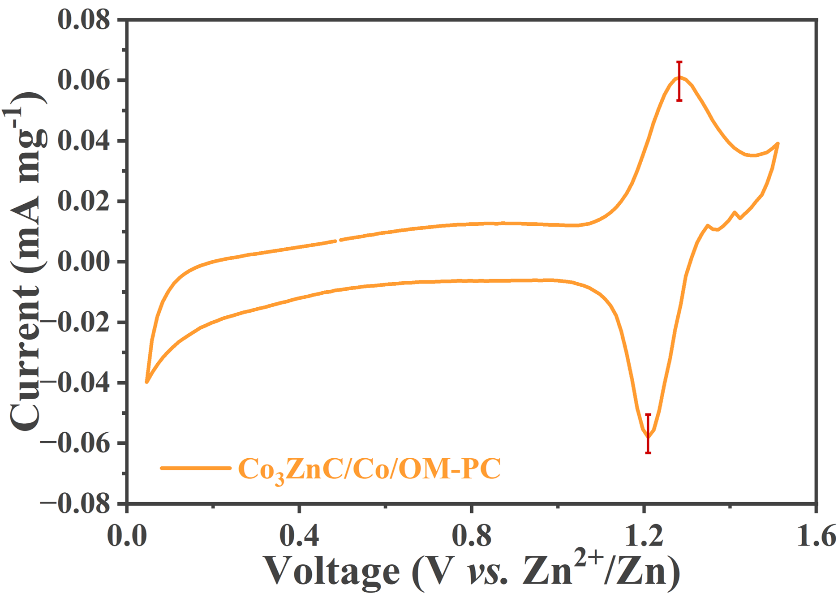


Figure S12 CV curve of an aqueous Zn-ion battery with the cathode of Co_3_ZnC/Co/OM-PC without loading S.


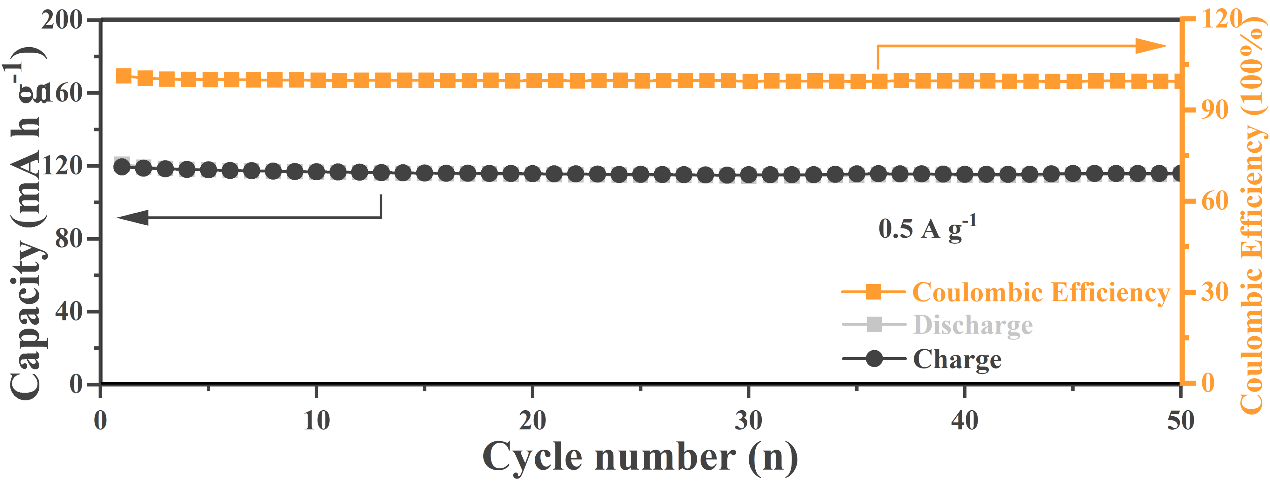


Figure S13 the discharge-charge cycle of Co_3_ZnC/Co/OM-PC cathode electrode at 0.5A g^-1^ with 2M Zn(OTf)_2_/ZnI_2_/G4/W electrolyte. (Capacity calculated based on the mass of iodine)


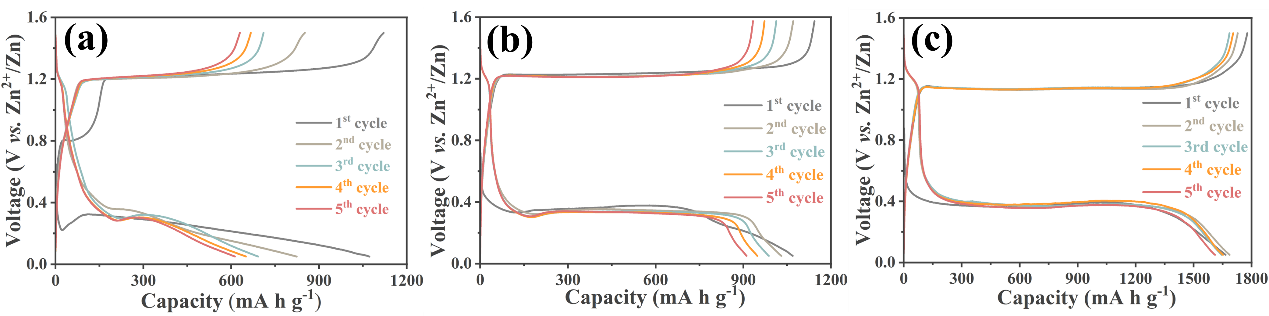


Figure S14 The first five cycles of GCD curves of S@PC (a), S@OM-PC (b) and S@Co_3_ZnC/Co/OM-PC (c). (S loading: 1.5-3.0 mg cm^-2^)


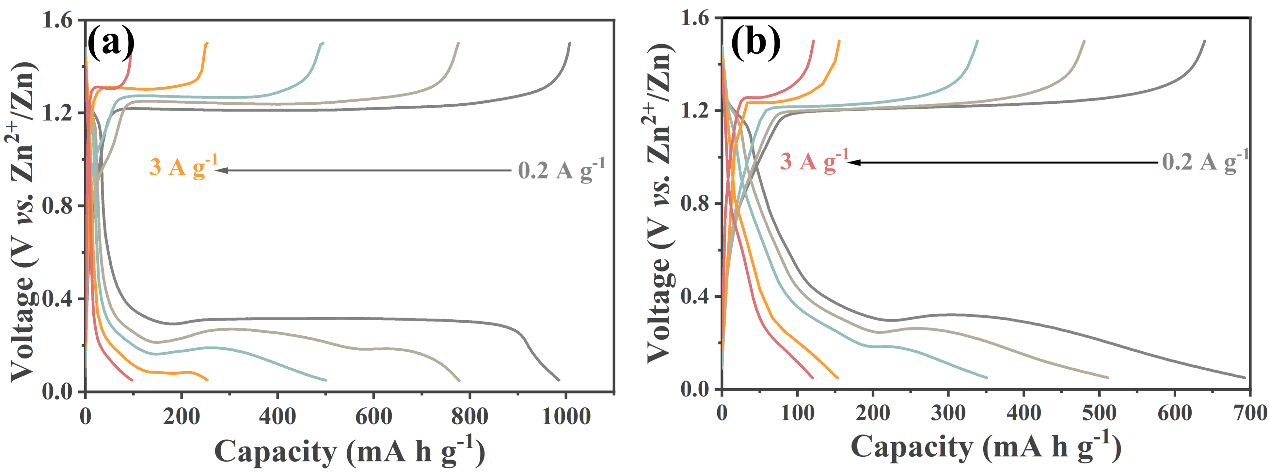


Figure S15 GCD curves at current density from 0.2 A g^-1^ to 3 A g^-1^ of S@OM-PC electrode (a) and S@PC (b). (S loading: 1.2-2.0 mg cm^-2^)


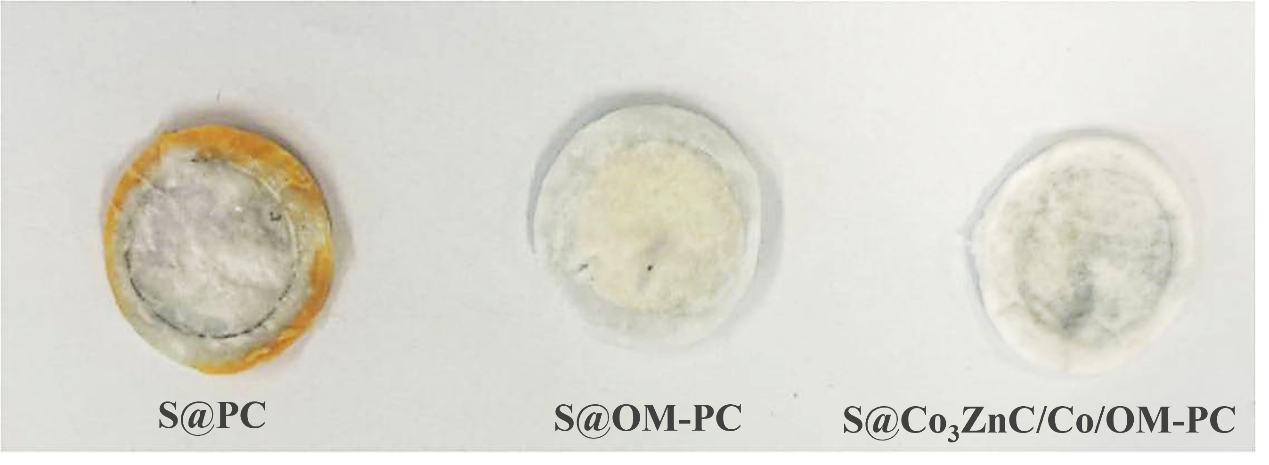


Figure S16 (a) The optical picture of the separator in aqueous Zn-S batteries with S@PC, S@OM-PC and S@Co_3_ZnC/Co/OM-PC after running 50 cycles.


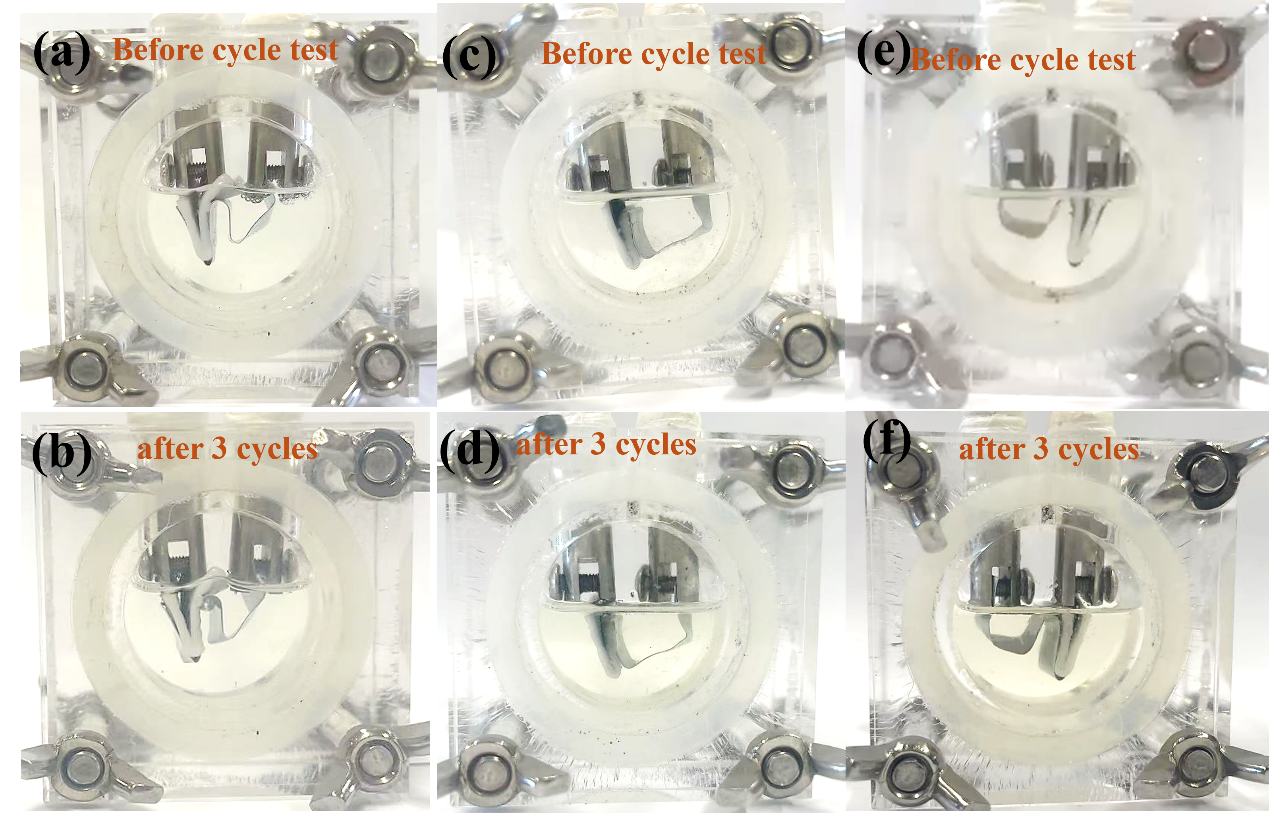


Figure S17 The optical picture of color change of the electrolyte (ZnOTf_2_/G4/W) (6mL) in which the S@Co_3_ZnC/Co/OM-PC, S@OM-PC and S@PC electrode undergo 3 cycles at 0.05 A g^-1^ (a, c and e) before (left) and (b, d and f) after (right) charge/discharge cycles, respectively.


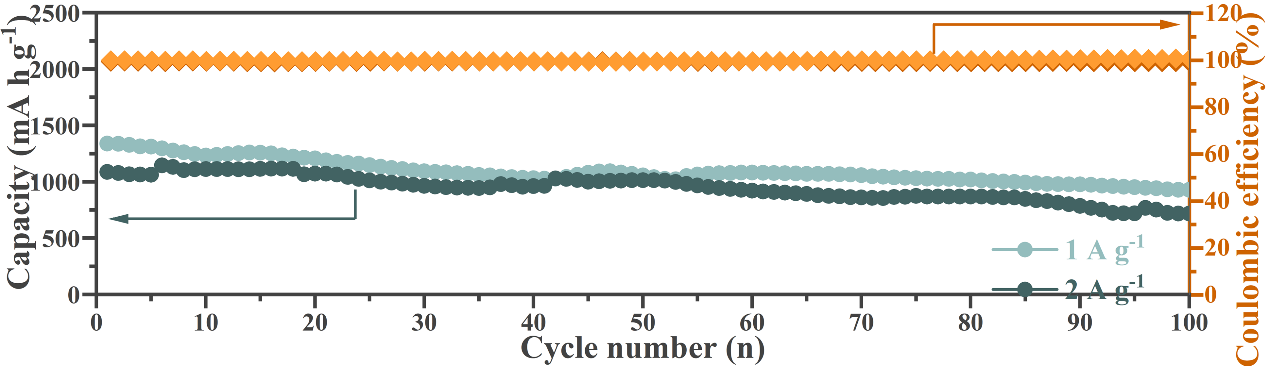


Figure S18 The Cycling performance of S@Co_3_ZnC/Co/OM-PC at 1 and 2 A g^-1^. (S loading: 1.5-2.3 mg cm^-2^).


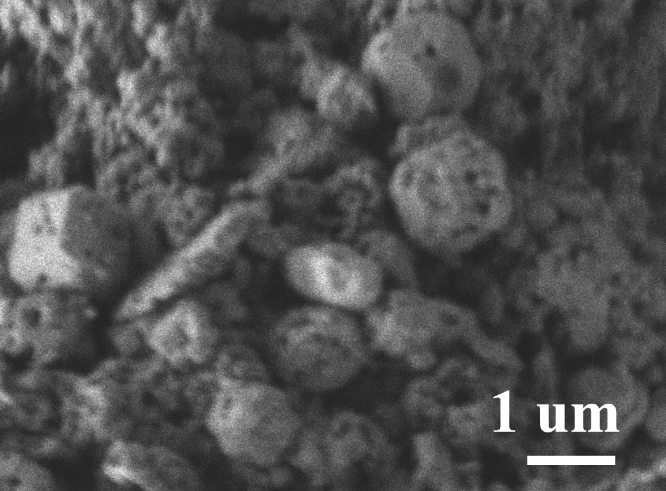


Figure S19 SEM of S@Co_3_ZnC/Co/OM-PC after 50 cycles at 0.5 A g^-1^.


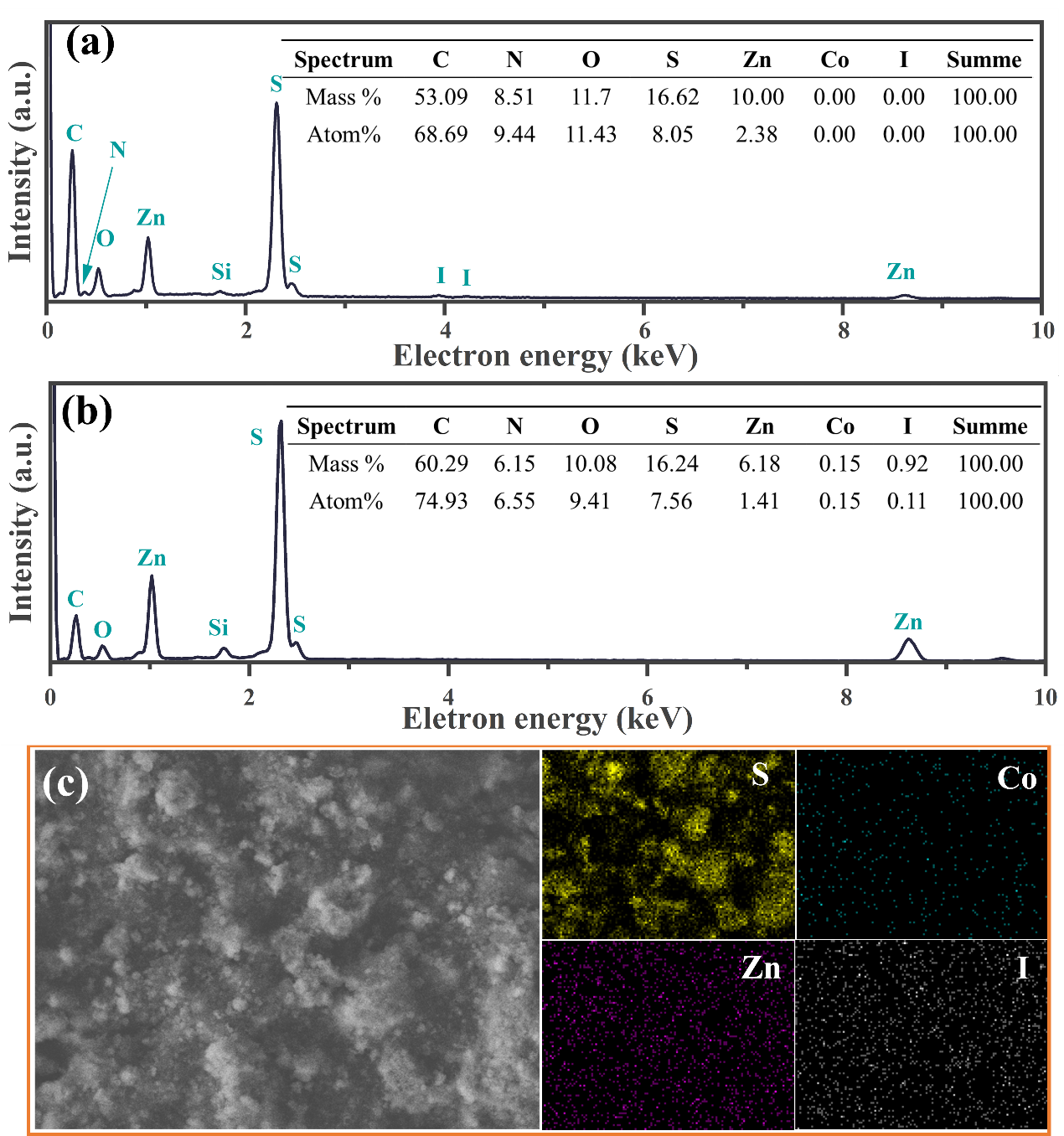


Figure S20 EDS spectra of the S@Co_3_ZnC/Co/OM-PC after soaking in electrolyte for 12h (a) and the S@Co_3_ZnC/Co/OM-PC after 50 cycles at 0.5A g^-1^ (b). (c) The EDS mapping of the S@Co_3_ZnC/Co/OM-PC after soaking in electrolyte for 12h.


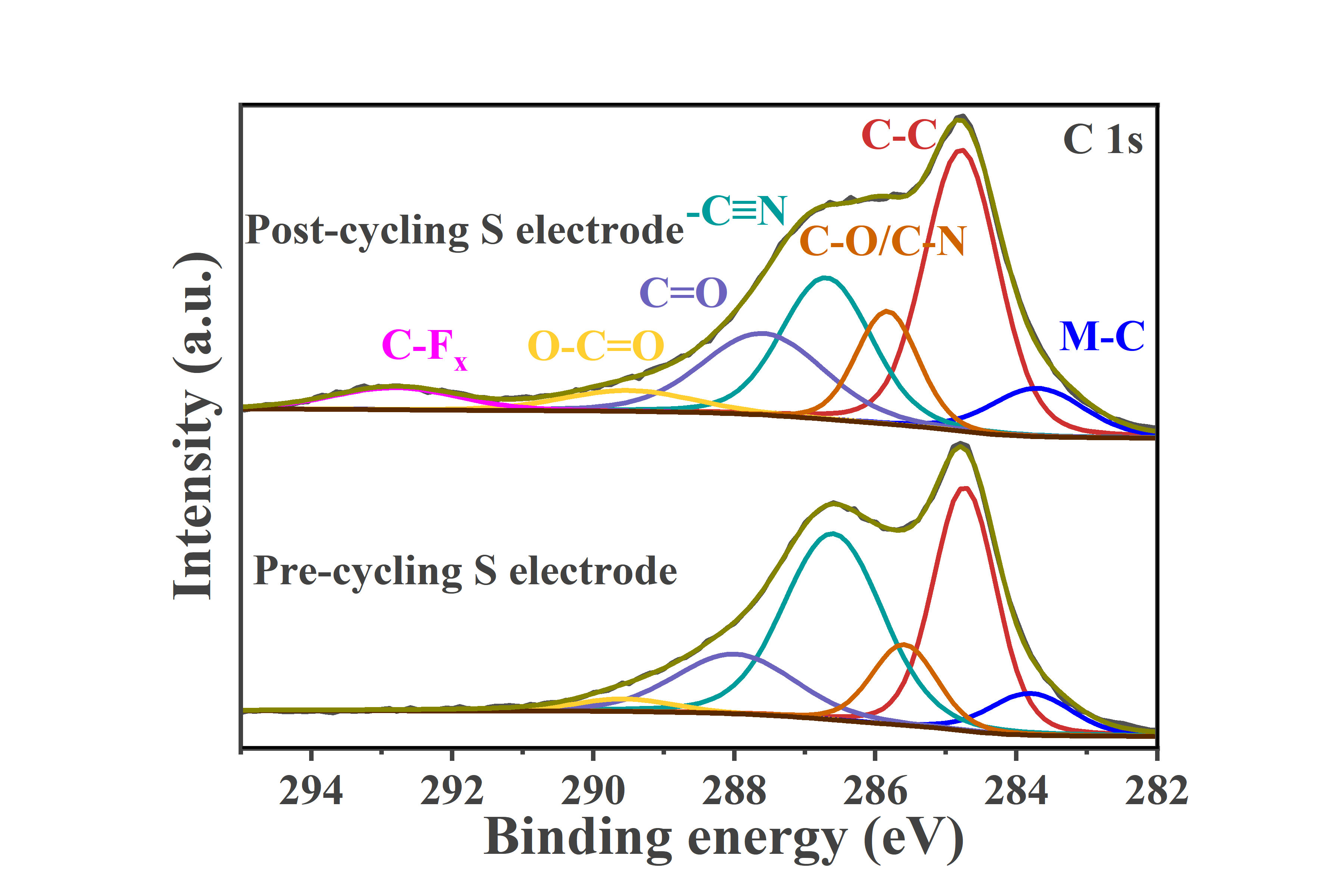


Figure S21 The high-resolution XPS spectra of C 1s of the S@Co_3_ZnC/Co/OM-PC after soaking in electrolyte for 12h (pre-cycling electrode) and the S@Co_3_ZnC/Co/OM-PC after 50 cycles at 0.5A g^-1^ (post-cycling electrode).


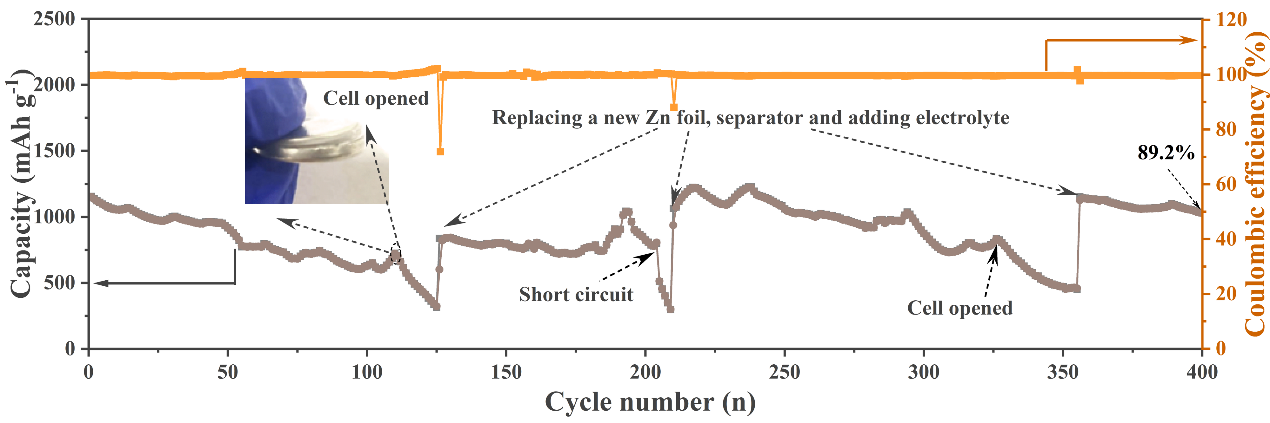


Figure S22 Cycling performance of S@Co_3_ZnC/Co/OM-PC at 3A g^-1^ with changing Zn anode and separator but without changing the S@Co_3_ZnC/Co/OM-PC cathode. (S loading: 1.75 mg cm^-2^).


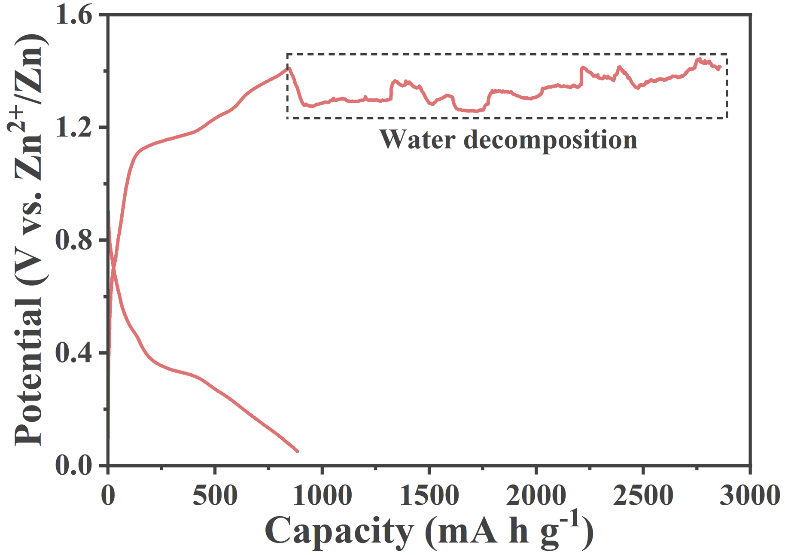


Figure S23 Charge and discharge curves of S@Co_3_ZnC/Co/OM-PC at a current density of 3 A g^-1^.


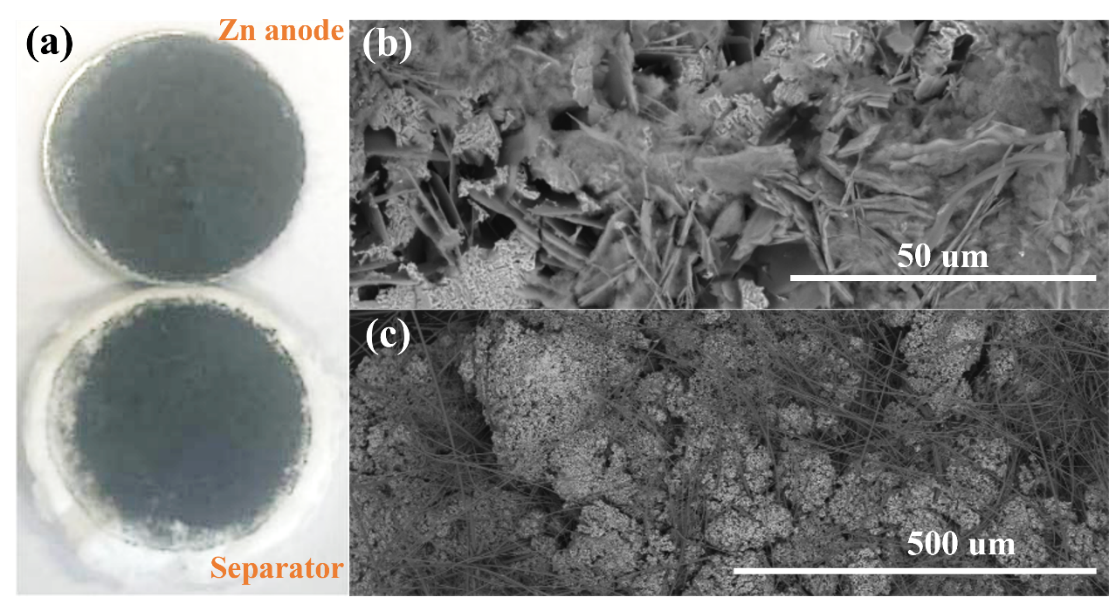


Figure 24 (a) The photo images of Zn anode (top) and separator (bottom) and the SEM images of the Zn dendrite on the (b) Zn anode and (c) the separator.


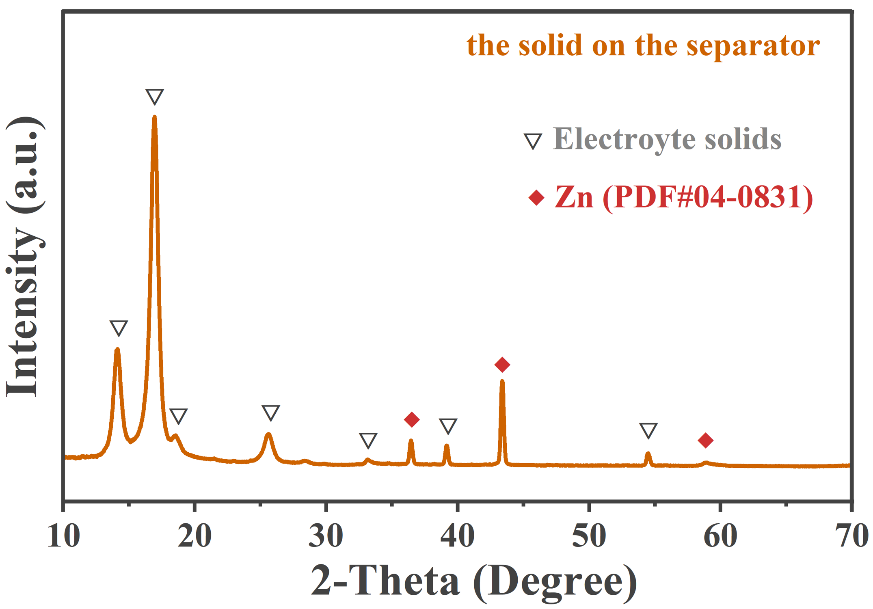


Figure S25 The XRD of the separator of the aqueous Zn-S batteries with S@Co_3_ZnC/Co/OM-PC after running 150 cycles.


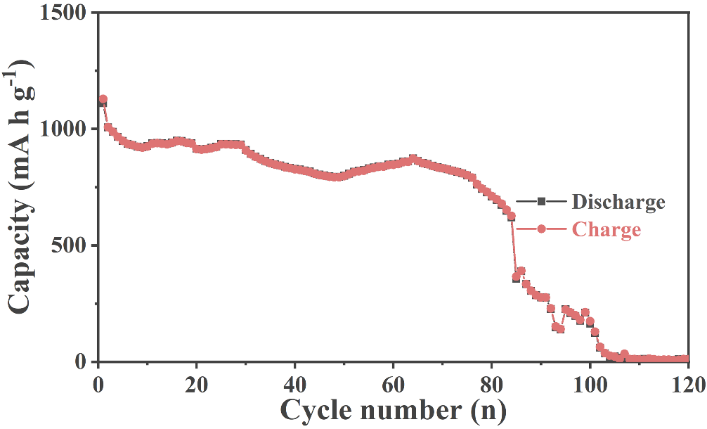


Figure S26 Cycling of S@Co_3_ZnC/Co/OM-PC at a current density of 3 A g^-1^.


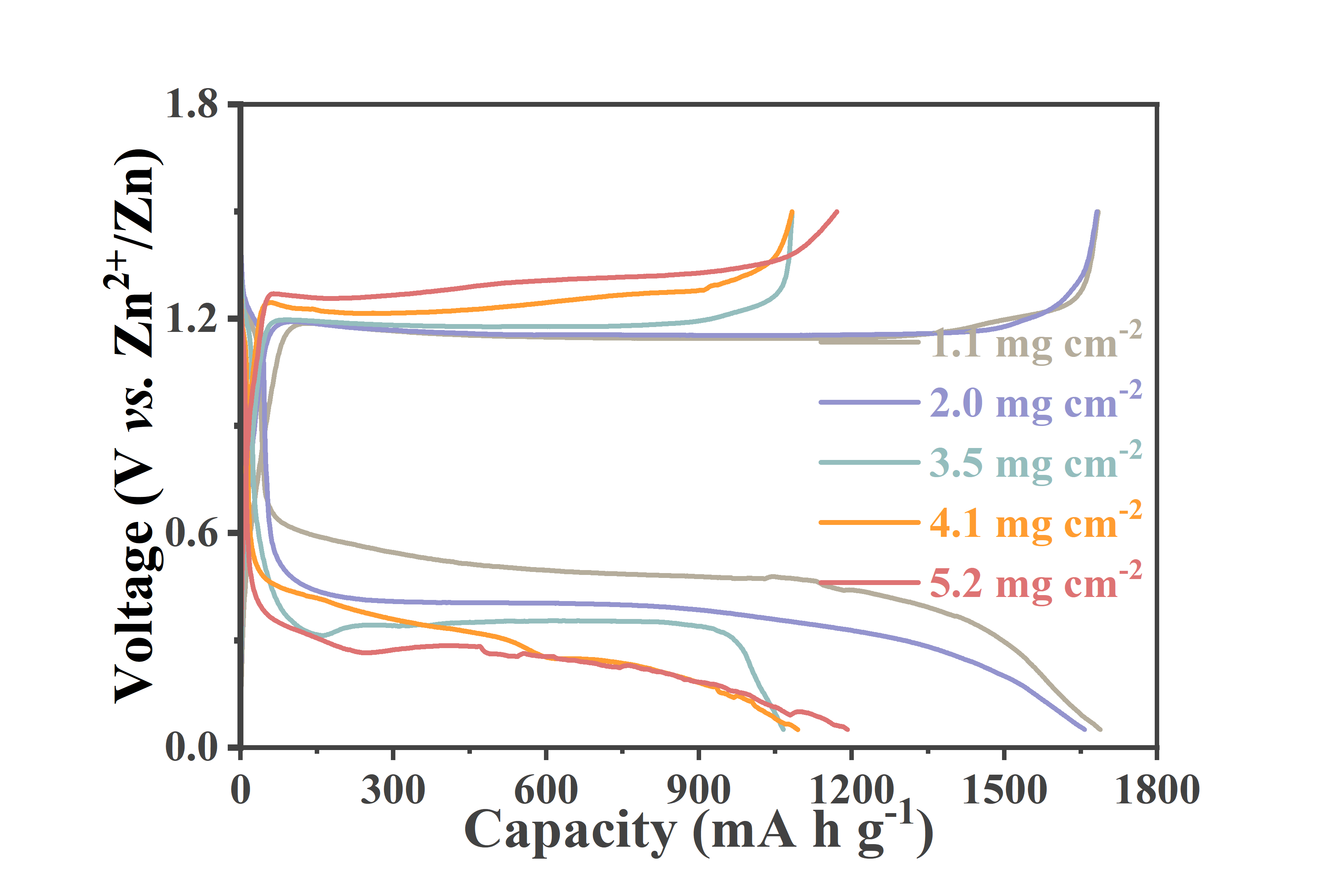


Figure S27 Charge and discharge curves of S@Co_3_ZnC/Co/OM-PC at a current density of 0.2 A g^-1^ with S loading of 1.1, 2.0, 3.5, 4.1 and 5.8 mg cm^-2^.


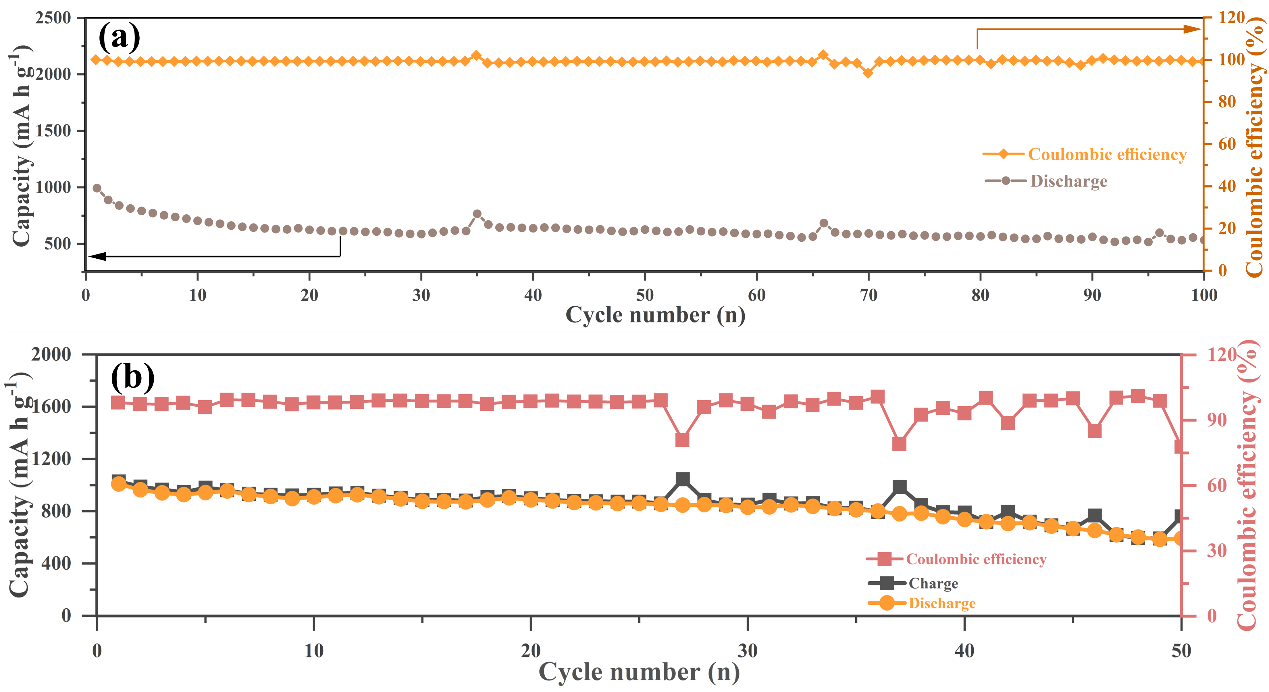


Figure S28 Cycling performance of the S@Co_3_ZnC/Co/OM-PC cathode at 0.2 A g^-1^. (a) S loading: 3.5 mg cm^-2^, (b) S loading: 4.1 mg cm^-2^.

Table S1 The ICP-MS results of Co and Zn in all the samples.

| Samples  Element | Co_3_ZnC/Co/OM-PC | OM-PC | PC |
| --- | --- | --- | --- |
| Co (ug/L) | 1.2 wt.% | - | - |
| Zn (ug/L) | 8.9 wt.% | 10.2 wt.% | 9.3 wt.% |

Table S2 the electrochemical performance and cell-testing conditions of aqueous zinc–sulfur studies.

| Catalysts | Capacity  (mA h g^-1^) | Discharge Voltage (versus Zn^2+^/Zn) | Voltage Hysteresis (versus Zn^2+^/Zn) | Electrolytes | Cycle stability | Ref. |
| --- | --- | --- | --- | --- | --- | --- |
| Aqueous Electrolyte | | | | | | |
| Phosphorus-doped carbon sheets (SeS_2_@PCS) | 1107 at 0.1 A/g | 0.74V at 0.1 A/g | 0.41V at 0.1 A/g | 1M ZnSO_4_ with I_2_ | 85% after 1000 cycles at 5A/g | ^2^ |
| Ketjen black (S@KB) | 1785 at 1 A/g | 0.45V at 1 A/g | 0.46V at 0.1 A/g | ZnSO_4_ + thiourea (TU) | a low decay rate of 0.11 % per cycle at 5A/g for 300 cycles | ^3^ |
| ZnS@CF | ~ 1050 at 2 A/g | ~ 0.6V at 0.1 A/g | 0.5V at 0.1 A/g | 3M ZnSO_4_ + iodinated thiourea (TUI) | 226 mAh g^−1^ after 300 cycles at 2 A/g | ^4^ |
| The porous carbon (S@NPC) | 1435 at 0.1 A/g | 0.26V at 0.5 A/g | 0.88V at 0.5A/g | 2M Zn(CF_3_SO_3_)_2_ with 50mM ZnI_2_ (EG/Water) | capacity retention of 70% after 250 cycles at 3 A/g | ^5^ |
| 3D N, P Co-Doped Carbon Foam (SeS_5.76_@3D-NPCF) | 1222 at 0.2 A/g; | 0.71V at 0.2 A/g | 0.43V at 0.2 A/g | 3M ZnSO_4_ with 0.1 wt% I_2_ | stable cycling of 75% capacity retention after 500 cycles at 4 A/g | ^6^ |
| Hollow carbon spheres (S@HCS) | 1140 at 0.5 A/g | ~ 0.5V at 0.5 A/g | 0.71V at 0.2 A/g | 2M Zn(OTf)_2_ with 0.05 wt% I_2_ (Water/G4) | retains over 70% capacity after 600 cycles at 4 A/g | ^7^ |
| Carbon Nanotubes Supported S (S@CNTs) | 1335 at 1 A/g | ~ 0.5V at 1A g | 0.8V at 1 A/g | 1M Zn(CH_3_COO)_2_ with 0.05 wt% I_2_ | After 50 cycles, the reversible capacity was retention of 85% | ^8^ |
| S@Ketjen black | 1295 at 0.2 A/g | 0.67V at 1 A/g | 0.42V at 1 A/g | 2M ZnSO_4_ | 5000 cycles with a high retention ratio of 94.5% at 8A/g | ^9^ |
| S@Ketjen Black | 1375 at 0.05A/g | 0.7V at 0.05A/g | - | 1 M ZnCl_2_ aqueous | - | ^10^ |
| S@CMK-3 | 788 at 0.2A/g | 0.46V at 0.2A/g | - | - | - | ^11^ |
| S@CNTs | 1116 at 0.1 A/g | - | 0.47 V at 0.2 A/g | 1 M Zn(CH_3_COO)_2_ electrolyte containing I_2_ (0.05 wt%) (water/PEG-400) | - | ^12^ |
| stable powder-Zn/indium (pZn/In) anode; CMK-3/S | 1630 at 0.1 C |  | 0.45V at 0.1 C | with 20 mM ZnI_2_ as electrolyte | Capacity decay from 1431 mAh g^−1^ to 670 mAh g^−1^ after 50 cycles. | ^13^ |
| Hydrogel Electrolyte | | | | | | |
| Polyaniline-wrapped sulfur | 1205 at 0.2 A/g | 0.58V at 0.2 A/g | 0.78 V at 0.2 A/g | PVA-ZnSO_4_ gel electrolyte | Capacity retention of 56% over low-current 50 cycles | ^14^ |
| activated carbon nanofiber (S@A-CNF) | 930 at 1 A/g | ~ 0.4V at 0.2 A/g | - | the PVA/zinc acetate dihydrate/W-EG solution with I_2_ | - | ^15^ |
| FeN_4_ single sites/ nitrogen-doped carbon/ carbon cloth (S@FeNC/NC/CC) | 1143 at 0.2 A/g | 0.69V at 0.2 A/g | 0.61V at 0.2 A/g | ZnSO_4_ gel (PVA) | A fade rate over 300 cycles of 0.141% per cycle | ^16^ |
| S@Ti_3_C_2_T_x_ composite | 772.7 at 0.5 A/g | ~ 0.57 V at 0.3 A/g | - | 1 M Zn(CH_3_COO) with 0.20 wt% I_2_ /30 wt% P123 pluronic block-copolymer | 91% capacity retention after 90° bending (500 cycles) | ^17^ |
| Conductive carbon black (S@C) | 846 at 0.5 A/g | ~ 0.6V at 0.5 A/g | ~ 0.87 V at 0.3 A/g | 5M ZnCl_2_+0.5m LiCl with 5% AN in deep eutectic solvent | Retains 33% capacity after 400 cycles at 1 A/g | ^18^ |
| sulfur-added activated  carbon (AC-S) | 675 at 0.25mA h/g | - | - | Zn(CH_3_COO) gel (PVA) with I_2_ (EG/Water 1/2) | Discharge capacity retention rate of 90% over 800 cycles | ^19^ |
| Co_3_ZnC/Co/OM-PC | 1685 at 0.2 A g-1 | ~ 0.67 at 0.2 A/g | ~ 0.67 V at 0.2 A/g | 2M Zn(OTf)_2_ with 1 wt% I_2_ (Water/G4) | capacity retention of 89.2% after 400 cycles at 3 A/g | This work |

Reference

1. Stein A.; Li F.; Denny NR.; Morphological-Control in Colloidal-Crystal-Templating of Inverse Opals, Hierarchical Structures, and Shaped particles. *Chemistry of Materials* 2008, 20(3),649-66.

2. Li, W.; Jing, X.; Ma, Y.; Chen, M.; Li, M.; Jiang, K.; Wang, D., Phosphorus-doped carbon sheets decorated with SeS_2_ as a cathode for aqueous Zn-SeS_2_ battery. *Chemical Engineering Journal* **2021,** *420*.

3. Chang, G.; Liu, J.; Hao, Y.; Huang, C.; Yang, Y.; Qian, Y.; Chen, X.; Tang, Q.; Hu, A., Bifunctional electrolyte additive with redox mediation and capacity contribution for sulfur cathode in aqueous Zn-S batteries. *Chemical Engineering Journal* **2023,** *457*.

4. Liu, D.; He, B.; Zhong, Y.; Chen, J.; Yuan, L.; Li, Z.; Huang, Y., A durable ZnS cathode for aqueous Zn-S batteries. *Nano Energy* **2022,** *101*.

5. Guo, Y.; Chua, R.; Chen, Y.; Cai, Y.; Tang, E. J. J.; Lim, J. J. N.; Tran, T. H.; Verma, V.; Wong, M. W.; Srinivasan, M., Hybrid Electrolyte Design for High-Performance Zinc-Sulfur Battery. *Small* **2023,** *19* (29), e2207133.

6. Li, W.; Ma, Y.; Li, P.; Jing, X.; Jiang, K.; Wang, D., Synergistic Effect between S and Se Enhancing the Electrochemical Behavior of SexSy in Aqueous Zn Metal Batteries. *Advanced Functional Materials* **2021,** *31* (20).

7. Yang, M.; Yan, Z.; Xiao, J.; Xin, W.; Zhang, L.; Peng, H.; Geng, Y.; Li, J.; Wang, Y.; Liu, L.; Zhu, Z., Boosting Cathode Activity and Anode Stability of Zn-S Batteries in Aqueous Media Through Cosolvent-Catalyst Synergy. *Angew Chem Int Ed Engl* **2022,** *61* (42), e202212666.

8. Li, W.; Wang, K.; Jiang, K., A Low Cost Aqueous Zn-S Battery Realizing Ultrahigh Energy Density. *Adv Sci (Weinh)* **2020,** *7* (23), 2000761.

9. Liu, J.; Ye, C.; Wu, H.; Jaroniec, M.; Qiao, S. Z., 2D Mesoporous Zincophilic Sieve for High-Rate Sulfur-Based Aqueous Zinc Batteries. *J Am Chem Soc* **2023,** *145* (9), 5384-5392.

10. Luo, L. W.; Zhang, C.; Wu, X.; Han, C.; Xu, Y.; Ji, X.; Jiang, J. X., A Zn-S aqueous primary battery with high energy and flat discharge plateau. *Chem Commun (Camb)* **2021,** *57* (77), 9918-9921.

11. Xu, Z.; Zhang, Y.; Gou, W.; Liu, M.; Sun, Y.; Han, X.; Sun, W.; Li, C., The key role of concentrated Zn(OTF)_2_ electrolyte in the performance of aqueous Zn-S batteries. *Chem Commun (Camb)* **2022,** *58* (58), 8145-8148.

12. Zhou, T.; Wan, H.; Liu, M.; Wu, Q.; Fan, Z.; Zhu, Y., Regulating uniform nucleation of ZnS enables low-polarized and high stable aqueous Zn–S batteries. *Materials Today Energy* **2022,** *27*.

13. Li, J.; Cheng, Z.; Li, Z.; Huang, Y., Rational design of zinc powder anode with high utilization and long cycle life for advanced aqueous Zn-S batteries. *Mater Horiz* **2023,** *10* (7), 2436-2444.

14. Zhang, H.; Shang, Z.; Luo, G.; Jiao, S.; Cao, R.; Chen, Q.; Lu, K., Redox Catalysis Promoted Activation of Sulfur Redox Chemistry for Energy-Dense Flexible Solid-State Zn-S Battery. *ACS Nano* **2022,** *16* (5), 7344-7351.

15. Amiri, A.; Sellers, R.; Naraghi, M.; Polycarpou, A. A., Multifunctional Quasi-Solid-State Zinc-Sulfur Battery. *ACS Nano* **2022**.

16. Zhang, W.; Wang, M.; Ma, J.; Zhang, H.; Fu, L.; Song, B.; Lu, S.; Lu, K., Bidirectional Atomic Iron Catalysis of Sulfur Redox Conversion in High‐Energy Flexible Zn-S Battery. *Advanced Functional Materials* **2023,** *33* (11).

17. Sonigara, K. K.; Vaghasiya, J. V.; Mayorga-Martinez, C. C.; Pumera, M., Flexible aqueous Zn-S battery based on an S-decorated Ti_3_C_2_T_x_ cathode. *npj 2D Materials and Applications* **2023,** *7* (1).

18. Cui, M.; Fei, J.; Mo, F.; Lei, H.; Huang, Y., Ultra-High-Capacity and Dendrite-Free Zinc-Sulfur Conversion Batteries Based on a Low-Cost Deep Eutectic Solvent. *ACS Appl Mater Interfaces* **2021,** *13* (46), 54981-54989.

19. Amiri, A.; Bashandeh, K.; Sellers, R.; Vaught, L.; Naraghi, M.; Polycarpou, A. A., Fully integrated design of a stretchable kirigami-inspired micro-sized zinc-sulfur battery. *Journal of Materials Chemistry A* **2023,** *11* (20), 10788-10797.
